# Staged identification of CAP in fever patients across epidemic environments: modeling & validation

**DOI:** 10.1038/s41598-025-29689-6

**Published:** 2025-12-18

**Authors:** Ziheng Gao, Tengfei Chen, Yanxiang Ha, Yifan Shi, Xiaolong Xu, Bo Li, Qingquan Liu

**Affiliations:** 1 Bejing University of Chinese Medicine, Beijing, 100029 China; 2 Beijing Hospital of Traditional Chinese Medicine, Capital Medical University, Beijing, 100010 China; 3 Beijing Institute of Chinese Medicine, Beijing, 100010 China; 4 Beijing Evidence-based Chinese Medicine Center, Beijing, 100010 China

**Keywords:** Community-acquired pneumonia, Machine learning, Risk prediction model, Traditional Chinese medicine, Epidemic environment, Experimental models of disease, Risk factors, Influenza virus, Respiratory tract diseases

## Abstract

Diagnosing community-acquired pneumonia (CAP) relies on costly imaging, posing challenges in resource-limited settings. Traditional tools focus on diagnostic tests for clinicians rather than patient use. Additionally, classification of subtypes in traditional Chinese medicine (TCM) lacks criteria. We developed a multimodal fusion model using machine learning algorithms and clinical variables from basic information, medical records, and lab tests to assess CAP risk in fever patients. The model integrates top-performing models via ensemble learning to predict pneumonia probability. We trained on 2,193 visits at Beijing Traditional Chinese Medicine Hospital’s fever clinic from Dec 2021 to Dec 2022, and validated on 300 visits from Jan to July 2024. Use unsupervised learning to classify subtypes. The training cohort included 1,781 CAP and similar patients, with 210 in the external validation cohort. CAPs were diagnosed via chest CT. The α model, based on pre-visit medical records, performed well (AUC_internal_=0.80, 95%CI 0.77–0.83; AUC_external_=0.80, 95%CI 0.71–0.87). The β model added four lab indicators, optimizing performance (AUC_internal_=0.93, 95%CI 0.92–0.95; AUC_external_=0.81, 95%CI 0.70–0.90). Two models were developed into online calculators. Latent class analysis distinguished Cold/Heat syndrome as subtypes. Despite the single-center, retrospective design, two final models performed good for identifying CAP across epidemic environments.

**Supplementary Information:**

The online version contains supplementary material available at 10.1038/s41598-025-29689-6.

## Introduction

Community-acquired pneumonia (CAP) is a common and frequently occurring respiratory infectious disease with high incidence and mortality rates^1,2^. Early identification of CAP is challenging, as its confirmation relies on expensive imaging equipment, such as chest CT^3,4^. But even X-ray examinations can only be interpreted by medical professionals. During sudden outbreaks, when medical resources are strained, medical congestion is particularly prominent in primary care clinics, emergency departments, and fever clinics. Moreover, pneumonia in the elderly population is clinically insidious and more difficult to identify early, leading to delayed treatment^5,6^.

The Pneumonia Severity Index (PSI) is a commonly used tool in clinical practice for clinicians and researchers to assess the severity of illness in hospitalized patients^7,8^. It includes many precise ancillary tests, making it difficult for patients to use and challenging to implement in rudimentary settings. Outpatients also struggle to complete all the required ancillary tests to calculate the score. Other scoring systems like CURB-65, A-DROP, and SMART-COP can rapidly assess pneumonia severity using a limited number of variables^8,9^. However, these assessments rely on significantly abnormal vital signssuch as respiratory rate, level of consciousness, blood pressure, and heart rateand specific ancillary tests (such as blood gas analysis, albumin, or blood urea nitrogen testing). They are primarily designed to quickly identify critically ill patients and provide emergency care, rather than to differentiate between pneumonia and non-pneumonia patients. Existing pneumonia prediction models often incorporate a wide range of clinical characteristic variables^10,11^, including high-cost chest CT imaging and specific biomarkers (such as IL-6, IL-10), aiming to accurately identify CAP patients, improve detection accuracy, and precisely identify pathogens. However, compared to further improving diagnostic accuracy, the ability to quickly, accurately, and cost-effectively identify pneumonia patients and enable stratified diagnosis and treatment during sudden outbreak responses is equally of great clinical value.

Additionally, the TCM treatment of CAP primarily focuses more on classifying syndromes by clinical symptoms than emphasizing etiology, a feature that has proven uniquely advantageous in the COVID-19 pandemic response^12,13^. Clinicians empirically classify CAP into Cold/Heat syndrome and select corresponding TCM interventions. Current TCM guideline only mentions the coexistence of “External Cold”“Internal Heat” without differentiation^14^. Therefore, the clinical classification of the two syndromes still lacks a standard.

During the COVID-19 pandemic, China implemented long-term and strict prevention and control policies from December 2019 to December 2022, creating a unique epidemiological environment. In this specialized social environment that effectively blocked the spread of the coronavirus, influenza viruses and other pathogens became the mainstream of respiratory viral infections^15,16^. After the end of these policies in December 2022, the Omicron variant of the coronavirus, rather than the Alpha or Delta variants, became widely prevalent. It has a lower proportion of severe cases and mortality rate, but stronger transmissibility and immune escape ability. The prevalent respiratory pathogens gradually shifted to mainly include the coronavirus, influenza virus, rhinovirus, and others^17,18^. It has become a new challenge for models to accurately identify pneumonia patients in different epidemiological environments, and even in the presence of new pathogens.

This study develops and validates a step-by-step CAP risk prediction model using multimodal data and machine learning across different epidemic settings. It aims to provide an easy-to-use, sensitive, and robust tool for early CAP detection. Additionally, it classifies CAP subtypes based on clinical symptoms to support TCM standardization.

## Materials and methods

### Materials

#### Data source

**Fig. 1:**
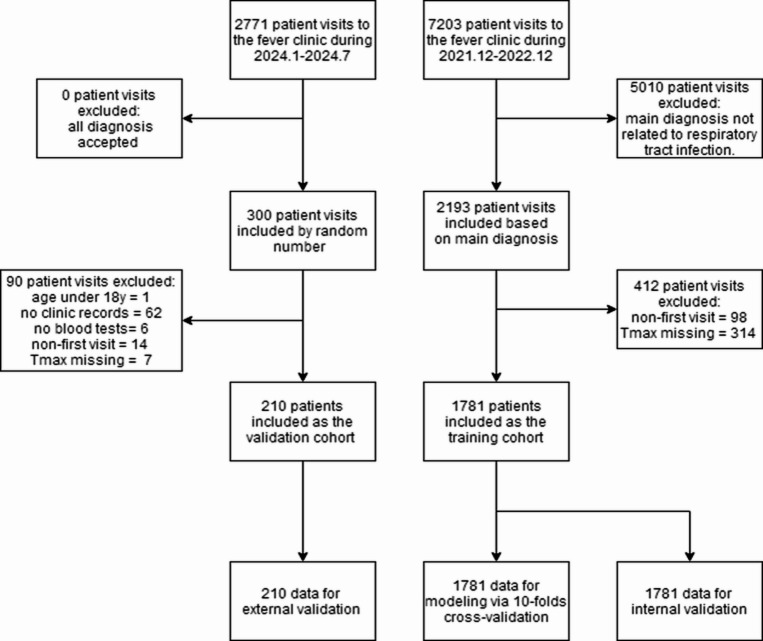
Flowchart of inclusion/exclusion of training/external validation cohort and composition of 10-fold cross/internal/external validation datasets.

The data used in this study were derived from two temporally and spatially independent cohorts of patients who visited the fever clinic of Beijing Hospital of Traditional Chinese Medicine (BHTCM), representing different epidemic environments. All data were anonymously extracted from the hospital information management system. From December 2021 to December 2022, 2,193 visits were selected from a total of 7,203 visits throughout the year based on disease diagnosis. After inclusion and exclusion criteria were applied, these visits formed the training cohort of 1,781 patients used for model development (also used for internal validation). From January to July 2024, 300 visits were randomly selected from a total of 2,771 visits over the six-month period (without disease diagnosis selection) to form the external validation cohort of 210 patients. The data from the two cohorts were kept separate, and the external validation cohort was used only after the model was completed (Fig. 1).

#### Inclusion criteria

We included patients with community-acquired pneumonia (CAP) and similar conditions (age over 18). The diagnoses of similar conditions needed to be related to respiratory tract infections, including acute upper respiratory tract infection, influenza, common cold, acute tracheitis/bronchitis, acute tonsillitis, acute pharyngitis, etc. All disease diagnoses were obtained from the outpatient information system. The clinical diagnosis and the CAP diagnostic criteria referenced in this study were as follows^3,4^:

(I) Onset in the community. (II) Presence of any one of the following manifestations related to pneumonia: (a) New onset of cough, sputum production, or worsening of pre-existing respiratory symptoms, with or without purulent sputum, chest pain, dyspnea, and hemoptysis; (b) Fever; (c) Physical signs of lung consolidation and/or auscultation of moist rales; (d) Peripheral blood leukocyte count > 10 × 10^9/L or < 4 × 10^9/L, with or without left shift. (III) Chest imaging showing new pulmonary infiltrates, lobar or segmental consolidation, ground-glass opacity, or interstitial changes, with or without pleural effusion.

#### Exclusion criteria

All patients included in the study were assessed against the following exclusion criteria: (I) Exclusion of follow-up medical records, retaining only the records of patients’ initial visits; (II) Exclusion of patients diagnosed with pneumonia who lack chest imaging evidence; (III) Exclusion of patients with conditions similar to severe CAP, such as pulmonary edema, diffuse alveolar hemorrhage, interstitial lung disease, etc.; (IV) Exclusion of patients who were transferred to another hospital or died on the day of their visit; (V) Exclusion of patients with missing important clinical variables (basic information, Tmax, number of days since onset of illness, disease diagnosis, etc.).

## Methods

### Disease diagnostic criteria

Community-acquired pneumonia (CAP) was diagnosed according to the latest relevant guideline criteria, and all patients with pneumonia in the study were required to have chest CT imaging to support the diagnosis. For practicality, we established the training cohort from patients with CAP and similar conditions rather than all patients who visited the clinic. Due to the different diagnostic perspectives, conditions such as “pulmonary infection” “pneumonia” “pneumonia caused by known viruses” “viral pneumonia” “bacterial pneumonia” and “fungal pneumonia” were considered clinically synonymous with CAP. Non-pneumonia patients were diagnosed with conditions such as “acute upper respiratory tract infection” “influenza caused by known viruses” “common cold” and “acute tonsillitis”, all of which were determined based on the standard ICD-10 disease names. Because of the differences in the epidemic environment, there were no patients with COVID-19 infection in the training cohort, and therefore no patients with CAP caused by COVID-19. Thus, diagnoses as “COVID-19 infection” and “COVID-19 pneumonia” were only present in the external validation cohort. Furthermore, we verified all patients in the database with an ICD-10 diagnosis of pneumonia to ensure they had supporting lung CT scans and were confirmed as pneumonia patients according to the CAP diagnostic criteria. Similarly, we reviewed all patients with lung CT examination results to confirm they did not meet any exclusion criteria specified in the CAP diagnostic guidelines, which might have led to a misdiagnosis of pneumonia. In other words, the confirmation of pneumonia based on lung CT support and fulfillment of the CAP diagnostic criteria served as the “gold standard” for patient selection in our study.

### Data collection & management

All patient data were anonymously extracted from the hospital information system, including basic information, medical records, laboratory test results, and chest CT examination results. Basic information included sex age, and date of visit, while medical records included chief complaints, medical history, past medical history, physical examination, and disease diagnosis. All patient data were anonymized (identifiable patient information was removed) and aggregated into an Excel database.

### Modeling & validation

In predictor selection, three strategies were compared comprehensively: (I) Directly using all extracted clinical feature variables; (II) Using only variables with significant differences between the pneumonia and non-pneumonia groups; (III) Using only important variables selected by Lasso regularization regression. Randomforest was used to model the training cohort, comparing the variable importance rankings among the three strategies and comparing the AUC values of the three models using 10-fold cross-validation. The strategy with the fewest variables was chosen as the predictor set. The same method was used to compare the variable importance rankings with interaction terms added, and the gain and necessity of adding interaction terms were assessed.

For modeling algorithms, traditional Logistic regression and five common machine learning algorithms were used (Logisticnet, Randomforest, XGBoost, AdaBoost, and CatBoost). To fully utilize the data, instead of using traditional random splitting methods(e.g., 7:3 training and validation set split), 10-fold cross-validation was conducted on the complete training set to obtain the best models and parameters for the six different algorithms. Among these, the “best” standard continues to be evaluated using the AUC value in subsequent stages. The six models were then internally validated and compared in the entire patient population. The temporally and spatially independent external validation set was used to evaluate the model performance in real clinical settings. The best-performing model among the six was determined as the final α model, with all predictors obtainable by patients before their visit. The Shapley Additive Explanations (SHAP) method was used to assess the impact of original variables on the output. For multimodal clinical data, a fusion modeling strategy was adopted by adding laboratory test-related variables to develop the β model. The β model was built on the probability obtained from the α model and included additional variables such as the neutrophil-to-lymphocyte ratio (NLR = NEU/LYM) and the C-reactive protein-to-platelet count ratio (CRP/PLT). These variables can be obtained through routine blood tests. The same six algorithms were used with 10-fold cross-validation for parameter tuning, modeling, and internal and external validation, with the best-performing β model selected. Both α/β models were developed into Shiny online web calculators for patients to assess pneumonia risk probability at different stages of their visit.

### Statistical analysis methods

This predictive modeling study employed a cross-sectional research design. Continuous variables were presented as medians and interquartile ranges, while categorical variables were described using counts and percentages. Normally distributed variables were compared using the two-sample t-test, non-normally distributed variables were compared using the Mann-Whitney U test, and categorical variables were compared using chi-square tests, Fisher’s exact tests, or Kruskal-Wallis tests as appropriate.

Missing values (with the proportion of missing values in each variable being less than 10%) were handled using multiple imputation by chained equations based on the random forest algorithm, with 10 imputations performed and the results combined according to Rubin’s rules. During model training, 10-fold cross-validation was utilized. The entire training set was randomly divided into 10 equal subsets, with one subset (10%) serving as the validation set and the remaining nine subsets (90%) as the training set for each iteration. This process was repeated 10 times, and the parameters that performed best in the validation set were selected as the optimal parameters for the model based on that algorithm. All p-values and confidence intervals were derived from two-sided tests, with a significance level set at 0.05. Bonferroni correction was applied to multiple testing to control the family-wise error rate (FWER) by using a more stringent significance level. Data cleaning, organization, and statistical analysis were conducted using RStudio (R 4.4.3) and Excel software. All R packages used have been described, and the main R codes had been provided also (Supplement).

### Methods’ technical review

Our study was based on past cross-sectional data from the fever clinic. We downloaded all the anonymized data from the Beijing Hospital of Traditional Chinese Medicine’s YiduCloud HIS database after obtaining approval from the hospital’s clinical research department, which reviewed our protocol and waived informed consent from the original patients. Results are reported according to the TRIPOD statement (Supplementary Table 8).

### Research support and funding

This study was funded by the National Natural Science Foundation of China (NNSFC 81774146) through the Beijing Hospital of Traditional Chinese Medicine. The sponsor had no role in the study. As its retrospective, anonymized, and non-intervention nature, the hospital’s ethics committee waived ethical approval during the model’s development, following the Declaration of Helsinki.

## Results

### Patient clinical characteristics

**Fig. 2:**
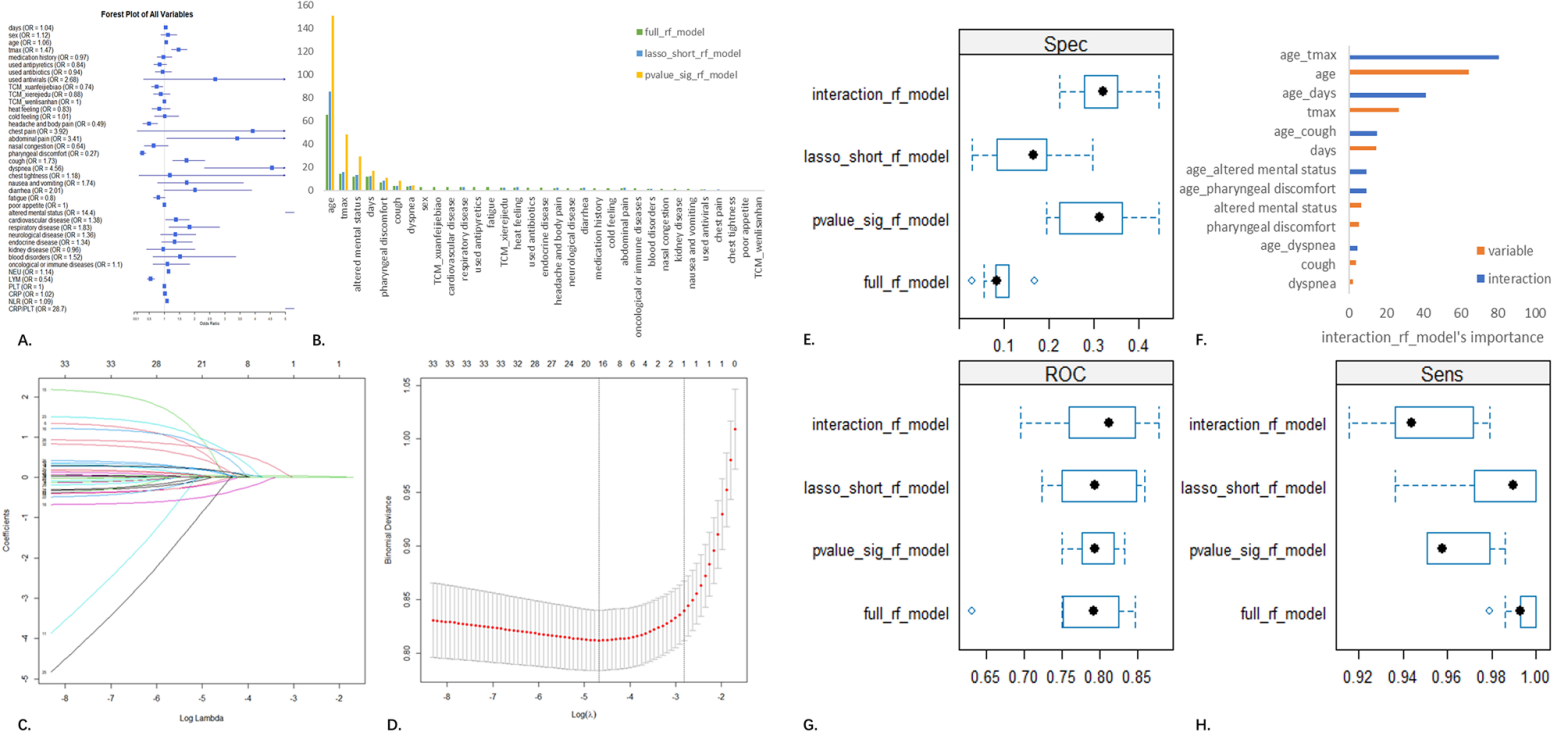
Evaluation plots for predictor selection strategies and interaction construction. (**A**) Forest plot of clinical variables; (**B**) Comparison of variable importance for three strategies selecting predictors; (**C,D**) Lasso regression plots; (**E,G,H**) Performance comparison of Randomforest models using three strategies; (**F**) Comparison of variable importance in Randomforest models after incorporating interaction terms.

We compared the clinical characteristics of patients with pneumonia and those without pneumonia in the training cohort (*N* = 362 vs. *N* = 1419) and calculated the odds ratios (OR) (Table 1). Similar comparison also conducted in the external validation cohort after modeling (Supplementary Table 1). Among the 33 variables derived from general information and medical records, seven variables—age, Tmax, days (since onset of illness), presence or worsening of pharyngeal discomfort, presence or worsening of cough, presence or worsening of dyspnea, and presence or worsening of altered mental status—remained significantly different between the groups after adjustment (all *P* < 0.001). The presence or worsening of headache and abdominal pain were only significantly different before adjustment (OR_hb_pain_=0.49, 95%CI 0.29–0.78, *P* = 0.002; OR_ab_pain_=3.41, 95%CI 1.07–10.5, *P* = 0.039). Regarding underlying diseases, we examined the differences between groups for cardiovascular disease, respiratory disease, neurological disease, kidney disease, blood disorders, and oncological/immune diseases. Only cardiovascular disease and respiratory disease showed significant differences before adjustment (OR_card_=1.38, 95%CI 1.04–1.82, *P* = 0.025 ; OR_resp_=1.83, 95%CI 1.15–2.83, *P* = 0.011), but these differences were not significant after adjustment. In terms of medication history, we compared the use of medications since the onset of illness. There were no significant differences in the use of antipyretics, antibiotics, or antiviral drugs. For traditional Chinese medicine (TCM), the use of TCM_XFJB was only significantly different before adjustment (OR_tcm_xfjb_=0.74, 95%CI 0.57–0.95, *P* = 0.019). In laboratory tests, we extracted six key clinical variables related to infection: neutrophil count (NEU), lymphocyte count (LYM), platelet count (PLT), C-reactive protein (CRP), neutrophil-to-lymphocyte ratio (NLR), and C-reactive protein-to-platelet count ratio (CRP/PLT). All these variables showed significant differences between groups after adjustment (all *P* < 0.001). In the hypothesis testing above, the Bonferroni correction was used to adjust the significance level to 0.05/33 + 6 ≈ 0.001. Among the three predictor selection strategies for the random forest model, the third strategy performed the best (AUC_I_=0.78; AUC_II_=0.78; AUC_III_=0.80). After comparing the variable importance rankings, the final α model used only the seven variables that remained significantly different between groups after multiple testing as predictors (Fig. 2).

**Table 1:** Comparison of characteristic between pneumonia and Non-Pneumonia in the training Cohort.

Characteristic			Non-pneumonia	Pneumonia	Odds ratio 95%CI	P value
			(*N* = 1419)	(*N* = 362)		
Basic Profile	Sex	Female = 0	713 (50.2%)	172 (47.5%)	1.12 [0.89–1.41]	0.354
		Male = 1	706 (49.8%)	190 (52.5%)		
	Age(y)		39.2 (17.2)	62.2 (22.2)	1.06 [1.05–1.06]	< 0.001^***^
	Days(d)		2.58 (4.05)	3.50 (4.49)	1.04 [1.02–1.07]	< 0.001^***^
	Tmax(℃)		38.1 (0.64)	38.3 (0.71)	1.47 [1.24–1.75]	< 0.001^***^
Past Medication History	Medication history	No = 0	430 (30.3%)	112 (30.9%)	0.97 [0.76–1.25]	0.864
		Yes = 1	989 (69.7%)	250 (69.1%)		
	Used antipyretics	No = 0	980 (69.1%)	263 (72.7%)	0.84 [0.65–1.08]	0.206
		Yes = 1	439 (30.9%)	99 (27.3%)		
	Used antibiotics	No = 0	1109 (78.2%)	287 (79.3%)	0.94 [0.70–1.24]	0.694
		Yes = 1	310 (21.8%)	75 (20.7%)		
	Used antivirals	No = 0	1416 (99.8%)	360 (99.4%)	2.68 [0.31–17.7]	0.269
		Yes = 1	3 (0.21%)	2 (0.55%)		
	TCM_XFJB	No = 0	923 (65.0%)	259 (71.5%)	0.74 [0.57–0.95]	0.023^*^
		Yes = 1	496 (35.0%)	103 (28.5%)		
	TCM_XRJD	No = 0	1161 (81.8%)	303 (83.7%)	0.88 [0.64–1.19]	0.448
		Yes = 1	258 (18.2%)	59 (16.3%)		
	TCM_WLSH	No = 0	1418 (99.9%)	362 (100%)	1	1
		Yes = 1	1 (0.07%)	0 (0.00%)		
Clinical Manifestation	Heat feeling	No = 0	153 (10.8%)	46 (12.7%)	0.83 [0.59–1.19]	0.345
		Yes = 1	1266 (89.2%)	316 (87.3%)		
	Cold feeling	No = 0	1279 (90.1%)	326 (90.1%)	1.01 [0.68–1.47]	1
		Yes = 1	140 (9.87%)	36 (9.94%)		
	Headache& body pain	No = 0	1273 (89.7%)	343 (94.8%)	0.49 [0.29–0.78]	0.004^**^
		Yes = 1	146 (10.3%)	19 (5.25%)		
	Chest pain	No = 0	1418 (99.9%)	361 (99.7%)	3.92 [0.10–153]	0.365
		Yes = 1	1 (0.07%)	1 (0.28%)		
	Abdominal pain	No = 0	1412 (99.5%)	356 (98.3%)	3.41 [1.07–10.5]	0.032^*^
		Yes = 1	7 (0.49%)	6 (1.66%)		
	Nasal congestion	No = 0	1340 (94.4%)	349 (96.4%)	0.64 [0.33–1.12]	0.167
		Yes = 1	79 (5.57%)	13 (3.59%)		
	Pharyngeal discomfort	No = 0	1040 (73.3%)	330 (91.2%)	0.27 [0.18–0.39]	< 0.001^***^
		Yes = 1	379 (26.7%)	32 (8.84%)		
	Cough	No = 0	1241 (87.5%)	290 (80.1%)	1.73 [1.27–2.34]	< 0.001^***^
		Yes = 1	178 (12.5%)	72 (19.9%)		
	Dyspnea	No = 0	1402 (98.8%)	343 (94.8%)	4.56 [2.33–8.99]	< 0.001^***^
		Yes = 1	17 (1.20%)	19 (5.25%)		
	Chest tightness	No = 0	1412 (99.5%)	360 (99.4%)	1.18 [0.16–5.06]	1
		Yes = 1	7 (0.49%)	2 (0.55%)		
	Nausea& vomiting	No = 0	1396 (98.4%)	352 (97.2%)	1.74 [0.78–3.61]	0.223
		Yes = 1	23 (1.62%)	10 (2.76%)		
	Diarrhea	No = 0	1393 (98.2%)	349 (96.4%)	2.01 [0.99–3.89]	0.066
		Yes = 1	26 (1.83%)	13 (3.59%)		
	Fatigue	No = 0	827 (58.3%)	230 (63.5%)	0.80 [0.63–1.02]	0.079
		Yes = 1	592 (41.7%)	132 (36.5%)		
	Poor appetite	No = 0	1406 (99.1%)	362 (100%)	1	1
		Yes = 1	13 (0.92%)	0 (0.00%)		
	Altered mental status	No = 0	1408 (99.2%)	325 (89.8%)	14.4 [7.49–30.1]	< 0.001^***^
		Yes = 1	11 (0.78%)	37 (10.2%)		
Underlying Disease	Cardiovascular disease	No = 0	1161 (81.8%)	277 (76.5%)	1.38 [1.04–1.82]	0.027^*^
		Yes = 1	258 (18.2%)	85 (23.5%)		
	Respiratory disease	No = 0	1352 (95.3%)	332 (91.7%)	1.83 [1.15–2.83]	0.011^*^
		Yes = 1	67 (4.72%)	30 (8.29%)		
	Neurological disease	No = 0	1324 (93.3%)	330 (91.2%)	1.36 [0.88–2.04]	0.193
		Yes = 1	95 (6.69%)	32 (8.84%)		
	Endocrine disease	No = 0	1295 (91.3%)	321 (88.7%)	1.34 [0.91–1.93]	0.157
		Yes = 1	124 (8.74%)	41 (11.3%)		
	Kidney disease	No = 0	1386 (97.7%)	354 (97.8%)	0.96 [0.41–2.01]	1
		Yes = 1	33 (2.33%)	8 (2.21%)		
	Blood disorders	No = 0	1398 (98.5%)	354 (97.8%)	1.52 [0.62–3.36]	0.455
		Yes = 1	21 (1.48%)	8 (2.21%)		
	Oncological/immune diseases	No = 0	1354 (95.4%)	344 (95.0%)	1.10 [0.62–1.84]	0.86
		Yes = 1	65 (4.58%)	18 (4.97%)		
Blood Test Results	NEU(*10^9^/L)		6.88 (3.62)	9.17 (5.00)	1.14 [1.11–1.17]	< 0.001^***^
	LYM(*10^9^/L)		1.35 (0.76)	1.12 (0.60)	0.54 [0.43–0.67]	< 0.001^***^
	NLR		6.43 (5.38)	11.9 (12.7)	1.09 [1.07–1.10]	< 0.001^***^
	PLT(*10^9^/L)		220 (62.7)	213 (78.2)	1.00 [1.00–1.00]	0.115
	CRP(mg/L)		22.3 (31.5)	66.5 (68.8)	1.02 [1.02–1.02]	< 0.001^***^
	CRP/PLT		0.11 (0.17)	0.36 (0.42)	28.7 [16.8–49.1]	< 0.001^***^

Mean(SD) ; n(%), *P < 0.05, **P < 0.01, ***P < 0.001.

### Interaction analysis

Noticing that there was a significant age difference between pneumonia and non-pneumonia patients in the training cohort (mean age 62.2 vs.39.2), which is consistent with clinical practice, we introduced interaction terms of age with six other variables that showed significant differences between the groups. This is because elderly CAP patients often have insidious onset of illness and are prone to neglecting their condition. We used the random forest algorithm to model and evaluate this strategy, and found that the model performance was quite satisfactory (AUC_interaction_=0.80). The six interaction terms with age that we constructed all had higher importance rankings than the original features, indicating the presence of interactions (Fig. 2). Given the vigilance required for the elderly, when integrating the β model, in addition to using the probability calculated from the α model as a new factor and incorporating laboratory test-derived variables, we once again included age as a predictor.

### Model performance evaluation

#### Discrimination performance comparison

**Fig. 3:**
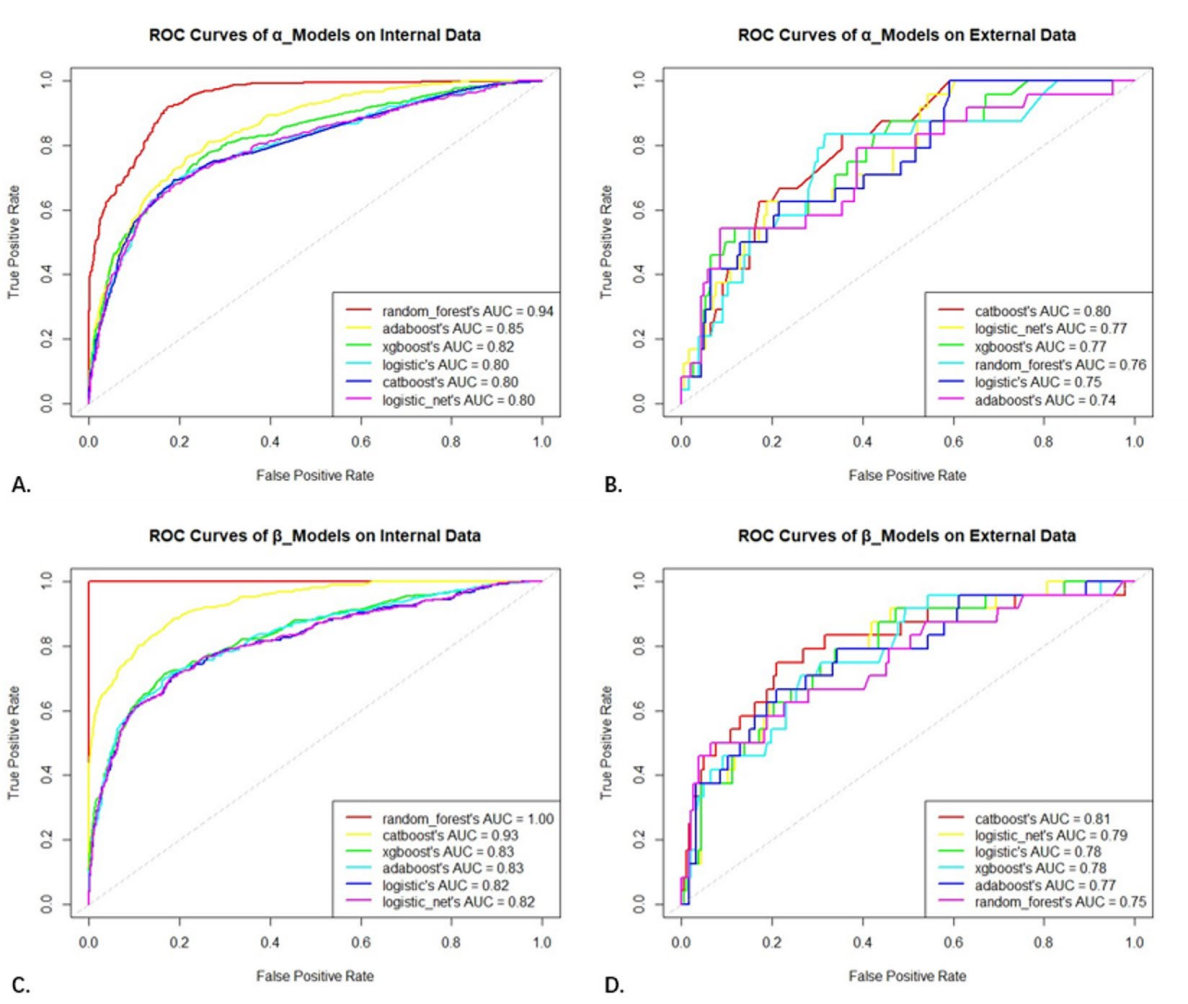
Comparison of ROC curves for 2 × 6  models. (**A,C**) Internal validation; (**B,D**) External validation.

“Whether diagnosed with pneumonia” as a binary classification prediction variable, its discrimination performance of the model was measured using the receiver operating characteristic curve (ROC) and the area under the curve (AUC), also known as the C-index. In the performance evaluation of the α model, comparison of six algorithms using 10-fold cross-validation suggested that five machine learning algorithms were superior to traditional Logistic regression, indicating practical value in selecting complex machine learning algorithms (Fig. 3). In the comparison of the optimal models of the six algorithms using the entire dataset as the internal validation set, the random forest model had the highest AUC (AUC_rf_=0.94, 95%CI 0.93–0.95), followed by the AdaBoost, XGBoost, and CatBoost models (AUC_ada_=0.85, 95%CI 0.83–0.87; AUC_xgb_=0.82, 95%CI 0.80–0.85; AUC_cat_=0.80, 95%CI 0.77–0.83). In the external validation set that was temporally and spatially independent, the six models were compared again, with the CatBoost model performing the best (AUC_cat_=0.80, 95%CI 0.71–0.87). The difference in AUC (|ΔAUC|) between internal and external validation was used to measure the robustness of the models, with CatBoost being the best (|ΔAUC|_cat_<0.01), while randomforest was the worst (|ΔAUC|_rf_=0.19). The Integrated Discrimination Improvement (IDI) was used to measure the improvement in the prediction probability of actual pneumonia patients by selecting the CatBoost algorithm (Figure S1). After a comprehensive comparison, the CatBoost model was selected as the final α model.

The β model also employed the same six algorithms and conducted internal/external validation, among other processes. In the comparison of models built with different algorithms, the final CatBoost model emerged as the winner (AUC_in_=0.93, 95%CI 0.92–0.95; AUC_ex_=0.81, 95%CI 0.70–0.90). When integrating the β model, the α model was incorporated into the modeling in the form of predicted probabilities as a new variable. As a result, the β model utilized the most clinical raw variables, covering multimodal clinical information including general information, medical records, and laboratory tests. The ROC curves of the α/β models were also compared in internal/external validation sets, with AUC calculated and DeLong tests conducted. It was found that the β model improved predictive performance in the training cohort compared to the α model (Z_in_=-13.91, *P* < 0.001), while the difference was not significant in the external validation cohort (Z_ex_=-0.32, *P* = 0.748) (Figure S2).

#### Detailed performance and classification thresholds

We further calculated the optimal cutoff and confusion matrices for the α/β models during internal and external validation. The cutoffs were selected based on the ROC curve, corresponding to the threshold that maximizes the sum of sensitivity and specificity. Compared to the α model, the β model had lower optimal cutoff during internal/external validation. However, the optimal cutoffs for the same model were similar across internal/external validation (Cutoff_α_in_=0.246, Cutoff_β_in_=0.145; Cutoff_α_ex_=0.222, Cutoff_β_ex_=0.151), reflecting the robustness of the α/β models under different epidemiological conditions. Both the training and external validation cohorts were imbalanced datasets (i.e., the number of non-pneumonia cases was several times higher than that of pneumonia cases). And the final model is intended for screening pneumonia patients, aiming to minimize missed diagnoses. Therefore, we focused on sensitivity, the Matthews Correlation Coefficient (MCC), and other indicators related to the confusion matrix. The Net Reclassification Improvement (NRI) analysis also indicated that the β model was superior (NRI_α/β_in_=0.20; NRI_α/β_ex_=0.33) (Table 2).

#### Robustness and generalization performance

Despite robustness measures (variable screening, 10-fold CV) ensuring stable AUCs, both models showed performance decay in external validation, with Model β loosing less. The purely real-world-derived external cohort (specifically tailored to clinical practice)—free from artificial inclusion/exclusion criteria—exposed generalization limitations when models confronting new fever patients. This shift was evidenced by the decline in CAP prevalence (internal’s 20% vs. external’s 11%), and we compared more of them (Supplementary Tables 2 to 7). As CAP cases decreased in both count and proportion, negative-class identification (Non-pneumonia) was challenged under intensified imbalance—manifested as specificity drop in Model α. Since AUC integrates sensitivity and specificity, our AUC-prioritized strategy likely preserved positive-class recognition, reflected in stable sensitivity and balanced accuracy. Macroscopically, the evolution to β model incorporates two interactions (NLR and CRP/PLT), ultimately enhancing its external generalization capability. ​ This suggests that ​stepwise CAP risk stratification, followed by ​basic blood tests for model-predicted positives​ to capture objective biomarker dynamics, could mitigate the limitations of α model.​.

**Table 2:** Comparison of performance based on the confusion matrix for the α/β Models.

Comparison	Count	Internal Validation		Count	External Validation	
	*N* = 1781	α_Model	β_Model	*N* = 210	α_Model	β_Model
True Positives (n(%))	*N* = 362 (20)	246 (68)	319 (88)	*N* = 24 (11)	19 (79)	17 (71)
False Negatives (n(%))		116 (32)	43 (12)		5 (21)	7 (29)
True Negatives (n(%))	*N* = 1419 (80)	1163 (82)	1156 (81)	*N* = 186 (89)	120 (65)	147 (79)
False Positives (n(%))		256 (18)	263 (19)		66 (35)	39 (21)
NRI_α/β_ (with Cutoff_β_)		NA	0.20		NA	0.33
IDI_α/β_		NA	0.26		NA	0.12
Matthews Correlation Coefficient		0.45	0.60		0.28	0.36
Balanced Accuracy		0.75	0.85		0.72	0.75
F1 Score		0.57	0.68		0.35	0.43
Sensitivity		0.68	0.88		0.79	0.71
Specificity		0.82	0.81		0.65	0.79
Accuracy		0.79	0.83		0.66	0.78

#### Calibration performance

**Fig. 4:**
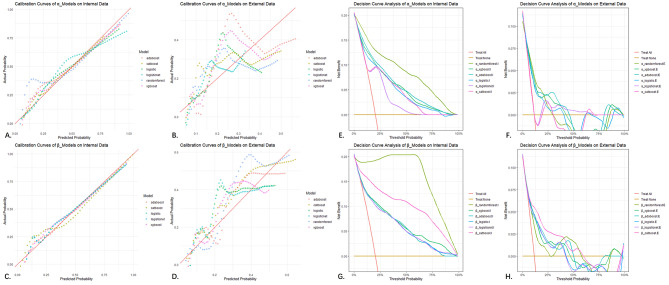
Comparison of calibration and decision curves for 2 × 6 models. (**A,C,E,G**) Internal validation; (**B,D,F,H**) External validation.

Calibration curves were drawn to assess the calibration performance. The closer the curve is to the diagonal line (from the bottom left to the top right), the better the calibration. The Brier score, which ranges from 0 to 1, is used to evaluate the accuracy of probability predictions, with lower values indicating better performance. During internal validation, the modeling processes of the α and β models using various algorithms were relatively close to the diagonal line. However, the random forest model in the β model exhibited overfitting (AUC_rf_=1), making it impossible to draw a calibration curveas it would overlap with the diagonal line. During external validation, the α/β models built using the CatBoost algorithm performed the best. Comparing the α and β models, all curves were found to be closer to the diagonal line, indicating that the integrated modeling strategy indeed provided added value. Comparing the Brier scores of internal/external validations also suggested that the β model was superior (Brier_α_in_=0.13, Brier_β_in_=0.08; Brier_α_ex_=0.11, Brier_β_ex_=0.08) (Fig. 4).

#### Clinical utility

Clinical decision curves were plotted to assess the clinical utility. By comparing the intersection relationships between the curves and two reference lines (treating all patients and treating no patients), the clinical benefit of applying the models was evaluated. During internal validation, the α/β models built by various algorithms were all above the reference lines across the entire probability range, indicating the potential clinical benefit of the models. The clinical net benefit value was obtained at the leftmost intersection point between the curve and the reference line (Net Benefit = 0.200), which means that approximately 20 more patients out of every 100 in the training cohort could receive correct diagnosis and treatment. During external validation, the α/β models built using the CatBoost algorithm still maintained curves above the two reference lines for most of the probability range. The β models built by all algorithms improved the clinical decision curves compared to the α models. Based on the final α/β models, approximately 11.3 more patients out of every 100 in the external validation cohort could receive correct diagnosis and treatment (Net Benefit = 0.113) (Fig. 4).

### Model interpretation and practical application

**Fig. 5:**
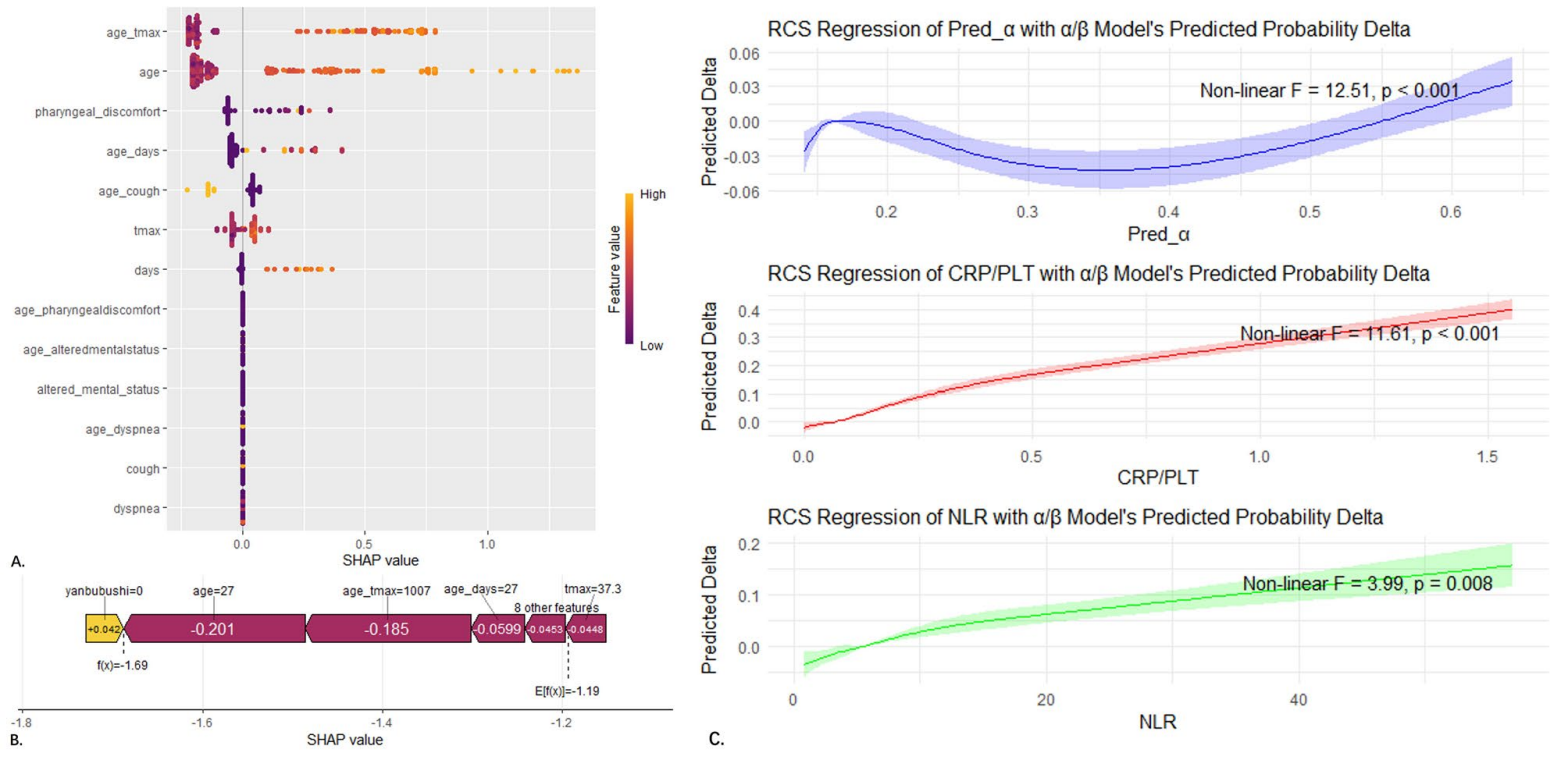
Series of interpretability analysis plots for the two best α/β models. (**A,B**) SHAP analysis for the α model; (**C**) RCS regression of the β model on the delta of predicted probability between the α and β.

Prior to modeling, we had already used the randomforest to calculate the importance ranking of variables. After determining the final model, we conducted SHAP interpretability analysis on the predictors of the α model again, finding that the SHAP value of the interaction term between age and Tmax was the highest, indicating that it is the most important for the α model. The β model incorporates additional test results and adjusts the predicted probability of the known α model. Therefore, we first calculated the difference in predicted probability between the β model and the α model (Probability Delta). We then used restricted cubic spline regression (RCS) on the predictors of the β model to elucidate the nonlinear relationships. Since age was highly collinear with the predicted probability of the α model, it was not included in the regression (Fig. 5). From the RCS regression plot, it can be seen that NLR (neutrophil-to-lymphocyte ratio) and CRP/PLT (C-reactive protein to platelet count ratio) are positively correlated with the probability difference (F_NLR_=11.61, *P* < 0.001; F_CRP/PLT_=-3.99, *P* = 0.008). An increase in these two factors indicates an upregulation of CAP risk. Regression analysis of the predicted probabilities of the α model shows that the β model makes smaller adjustments to extreme predicted probabilities (close to 0%or 100%) but downregulates most of the predicted probabilities. Both α/β models have been transformed into online web-based calculators using the Shiny package in RStudio. By directly entering clinical features and calculating the probabilities before and during the fever clinical visit, patients can autonomously assess the CAP risk (https://xjbqxmbt.shinyapps.io/shinyapps_PRO/*).*

### Identification of cap’s clinical subtypes

**Fig. 6:**
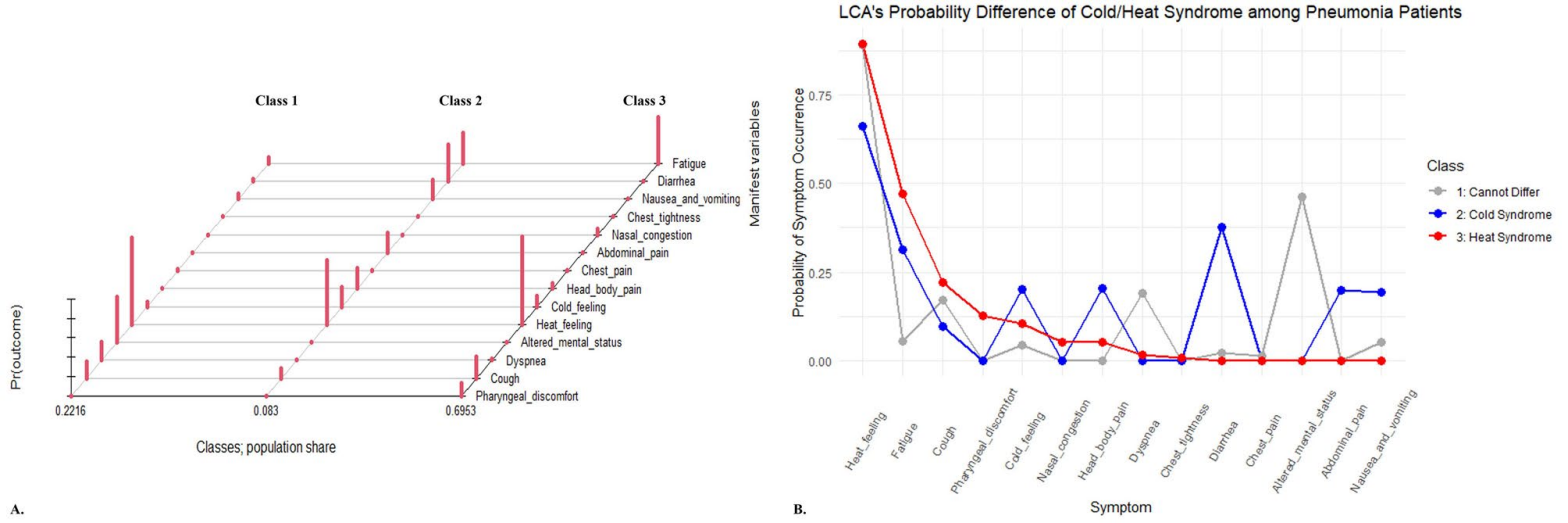
Series of latent class analysis charts for automatic subtype classification of CAP. (**A**) LCA plot of three latent classes containing the cold/heat syndrome; (**B**) LCA’s Probability differences between cold/heat syndrome.

After successfully developing the CAP risk prediction model, latent class analysis (LCA), an unsupervised machine learning method, was used to automatically distinguish clinical subtypes in 362 CAP patients only based on the probability distribution of clinical manifestations (Fig. 6). With setted latent classes number of three, LCA identified three clinically meaningful categories: (I) Class 1 (*N* = 48) with more severe illness, presenting with altered mental status and dyspnea, which are easily detectable and clearly related to pulmonary infection. These patients could not be further classified by symptoms due to the mental problem. (II) Class 2 (*N* = 22) presenting with cold feeling, headach& body pain, nausea& vomiting, abdominal pain, and diarrhea, consistent with the TCM concept of Cold Syndrome. (III) Class 3 (*N* = 292) presenting with heat feeling, fatigue, cough, pharyngeal discomfort, and nasal congestion, consistent with the TCM concept of Heat Syndrome. The predicted CAP risk probabilities were compared across the three classes. Class 1 had higher risk probabilities (Pred_α_1_ = 0.57 ± 0.09; Pred_β_1_ = 0.82 ± 0.18; W_α_=2851.5, *P* < 0.001; W_β_=2931, *P* < 0.001). Class 2/3 had similar risk (Pred_α_3_ = 0.38 ± 0.18, Pred_β_3_ = 0.52 ± 0.28; Pred_α2 = 0.33 ± 0.20, Pred_β_2_ = 0.49 ± 0.34; W_α_=2685, *P* = 0.199; W_β_=3078, *P* = 0.745). This confirms that the model can not only identify CAP patients, but also distinguish disease severity based on risk probabilities.

## Discussion

We explored the risk factors for CAP within the training cohort based on a cross-sectional study design framework. Some variables that were significant before adjustment (TCM_XFJB, Headache & body pain, Abdominal pain, Cardiovascular disease, Respiratory disease) were not included in the α model, but somes may still be potential risk factors. For example, underlying cardiovascular and respiratory diseases imply poorer cardiopulmonary functional reserve, which is associated with worse prognosis when facing respiratory infections. Guidelines for heart failure and COPD also indicate that infection is an important trigger for the acute exacerbation of these underlying diseases, and the two may interact causally in the overall progression^19,20^. Our model is designed to screen high-risk CAP patients in large cohorts of respiratory/fever patients, not for disease endpoints. Thus, assessing comorbidities after initial imaging confirmation is feasible. From a clinical perspective, abdominal pain is not directly associated with CAP. The wide confidence interval of OR is due to the small number of positive cases and large group sizes. However, some case reports suggest that pulmonary infections can cause abdominal pain, indicating a more severe condition, explained by the involvement of the diaphragm and gastrointestinal dysfunction due to systemic inflammatory response^21,22^. It is recommended that clinicians still pay attention to abdominal pain as an auxiliary judgment.

In this study, we aimed to develop a screening tool with as few features as possible, hence only seven clinical features with significant differences between groups were ultimately selected as predictors for the α model. Among them, altered mental status (OR = 14, 95%CI 7.49–30.1) and dyspnea (OR = 4.56, 95%CI 2.33–8.99) had very high odds ratios (OR). However, in the variable importance assessment based on randomforest and the SHAP analysis of the CatBoost model, the rankings of these two variables and their interaction terms with age were relatively low. In contrast, age (OR = 1.06, 95%CI 1.05–1.06), Tmax (OR = 1.47, 95%CI 1.24–1.75), and their interaction term were ranked higher. This indicates that machine learning algorithms do not rely on extreme clinical feature differences between groups, but can effectively capture the impact of common clinical features and their interactions on CAP risk. Therefore, even in external validation across different epidemic environments, the model still has good generalization ability. Clinically, altered mental status and dyspnea are key signs of severe CAP, easily alerting patients and doctors. However, mild symptoms make CAP risk hard to estimate, often delaying diagnosis and treatment, especially in elderly patients. Our model highlights the interaction between age and other factors, emphasizing the predictive value of mild symptoms like Tmax, days since onset of illness, cough, and pharyngeal discomfort in elderly patients. This helps dynamically assess and detect CAP early in this high-risk group.

When considering the predictors such as the number of days since the onset of illness (OR = 1.04, 95% CI 1.02–1.07), pharyngeal discomfort (OR = 0.27, 95% CI 0.18–0.39), and cough (OR = 1.73, 95% CI 1.27–2.34), in combination with other clinical features like headache& body pain (OR = 0.49, 95% CI 0.29–0.78), abdominal pain (OR = 3.41, 95% CI 1.07–10.5), and the use of TCM_XFJB (OR = 0.74, 95% CI 0.57–0.95), the differences in clinical features between groups align with the natural course of respiratory infectious diseases caused by pathogens. Referring to guidelines for common pathogens such as COVID-19 and influenza^23,24^, the progression from mild to moderate/severe/critical conditions is often marked by the migration of the infection from the upper respiratory tract to the lower. Latent class analysis (LCA) was conducted to further explore whether these clinical symptoms can distinguish patients (Figure S3). This transition is frequently described in traditional Chinese medicine theory as the progression from “Superficial syndromes” (headache& body pain, pharyngeal discomfort) to “Interior syndromes” (cough, abdominal pain, dyspnea, altered mental status) (Supplementary Table 9). This may explain why the use of TCM_XFJB (which are indicated for superficial syndromes) could be a potential protective factor against CAP. In other medication comparisons, no significant differences were found in the use of antipyretics, antibiotics, and antiviral drugs.This may reflect potential misuse, as these drugs can be obtained without clear diagnoses in community settings.Their preemptive use without confirmed indications does not reduce CAP risk.The single-center study design may introduce selection bias, and future multicenter studies are needed.

As an exploration of CAP diagnosis based on clinical symptoms, we identified three clinical subtypes, two of which align with TCM’s cold and heat syndromes. These syndromes determine different TCM treatments: “Warming the Cold” or “Clearing the Heat” (Supplementary Table 9). However, there are no standard criteria to classify the two in CAP. Therefore, we used unsupervised learning (latent class analysis) without adding labels manually. Class 1 represented severe cases with noticeable altered mental status and dyspnea. Class 2 and Class 3, which accounted for the majority (86.7%) of pneumonia patients, had CT evidence of pneumonia but lacked severe pulmonary infection symptoms. Compared to current assessment tools like PSI and CURB-65 that rely heavily on extensive tests or significant abnormal signs, our model (α/β) effectively identified these patients and reflected disease severity through predicted probabilities.

In the β model, we adjusted the predicted probabilities of the α model using NLR and CRP/PLT based on clinical experience. The NLR, calculated as the ratio of NEU to LYM in peripheral blood, is a biomarker that reflects both the innate immune response (mediated by NEU) and adaptive immunity (supported by LYM)^25–27^. An elevated NLR is often associated with inflammation, tissue damage, and a systemic inflammatory response, as neutrophils increase and lymphocytes decrease during infection or immune suppression. CRP/PLT is a composite index proposed by us based on clinical practice. It serves as an initial diagnostic marker in studies on neonatal pneumonia and sepsis^28^. However, research on adult pneumonia related to this index is rather limited and not very satisfactory^29–31^. CRP levels acutely rise in bacterial infections and severe viral infections, while PLT can significantly decrease due to inflammation or coagulation consumption, leading to a higher CRP/PLT. These two indicators, were chosen because they reflect the interplay of inflammation, immunity, and coagulation, which are central to the progression of respiratory infections from mild to severe^32–35^. In the β model, increases in NLR and CRP/PLT both imply an upregulation of the predicted CAP risk. According to the RCS regression, these two factors can bring about a maximum increase of approximately 10% and 40% in the predicted probability. In this study, only initial ancillary tests (complete blood count + CRP, chest CT) were used. Future work could develop more comprehensive dynamic prediction models incorporating imaging data and multiple follow-up test results to cover the entire disease course of respiratory infections.

The final α/β model, as a convenient online CAP screening tool, utilized a limited number of clinical features.The use of machine learning, multimodal information, and fusion strategies proved successful. Given the complexity of interpreting machine learning algorithms, this study supplemented the analysis with SHAP and RCS regression to evaluate the contribution of each predictor. Additionally, LCA was conducted to explore the natural classification of CAP clinical subtypes, laying the groundwork for future research.

## Conclusion

This study developed and externally validated two prediction models calculating the probability of confirmed CAP in a phased and stepwise manner. The final α/β models demonstrated satisfactory predictive performance and represent a new tool for assessing CAP risk in the population of febrile patients. The classification of CAP clinical subtypes corroborates the existing TCM experience in distinguishing Cold/Heat syndromes, providing support for future standardization. The single-center, retrospective design of this study is a limitation; future model updates and improvement studies should incorporate multicenter, prospective designs.

## Supplementary Information

Below is the link to the electronic supplementary material.

Supplementary Material 1

Supplementary Material 2

Supplementary Material 3

## Data Availability

The datasets generated and/or analyzed during the current study are available for scientific use after the corresponding authors upon reasonable request. We have included the primary research code and key processed datasets in the supplementary material. When aligned with research purposes, anyone may request the specific contents of lung CT imaging diagnoses for all pneumonia patients from the corresponding author.
