# Supplementary material for: Staged identification of CAP in fever patients across epidemic environments: modeling & validation
Source: Sci Rep. 2025 Dec 18;16:258. doi: 10.1038/s41598-025-29689-6 (PMC12770393; doi:10.1038/s41598-025-29689-6)
Supplement: Supplementary file 3 — Supplementary Material 3 [file 41598_2025_29689_MOESM3_ESM.docx]

**Staged Identification of CAP in Fever Patients Across Epidemic Environments: Modeling &Validation**

**Gao Ziheng, Chen Tengfei, Ha Yanxiang, Shi Yifan, Xu Xiaolong, Li Bo, Liu Qingquan**

**1. Modeling Sample Size Calculation**

**2. R Packages Used in Work**

**3. External Validation Cohort’s Clinical Characteristics**

**Supplementary Table 1** Comparison of Characteristics between Pneumonia and Non-Pneumonia in the External Validation Cohort

**Supplementary Table 2** Comparison of Characteristics between Internal/External Validation Cohorts

**Supplementary Table 3** Comparison of Characteristics between Pneumonia Patients in the Internal/External Validation Cohorts

**Supplementary Table 4** Comparison of Characteristics between Non-Pneumonia Patients in the Internal/External Validation Cohorts

**Supplementary Table 5** Comparison of Characteristics between COVID-19 and Non-COVID-19 in the External Validation Cohort

**Supplementary Table 6** Comparison of Characteristics between COVID-19 and the Internal Validation Cohort (surely non-COVID-19)

**Supplementary Table 7** Comparison of Characteristics between non-COVID-19 and the Internal Validation Cohort

**Figure S1** The Heatmap of IDI Analysis for the α/β Models in External Validation

**Figure S2** Comparison of the ROC Curves for the Final α/β Models

**4. Discussion of CAP Diagnostic Criteria**

**Figure S3** Series of Latent Class Analysis Charts for Automatic Subtype Classification of Internal Training Cohort

**Figure S4** The Correlation Heatmap among Clinical Variables in the Training Cohort

**5. Supplementary Calibration Figures and Models’ Performance across Subgroups**

**Figure S5** Calibration Curves of α_Models on Internal Dataset between 6 Algorithms

**Figure S6** Calibration Curves of α_Models on External Dataset between 6 Algorithms

**Figure S7** Calibration Curves of β_Models on Internal Dataset between 5 Algorithms

**Figure S8** Calibration Curves of β_Models on External Dataset between 6 Algorithms

**Figure S9** Final Models’ ROC Curves Across Sex Subgroups

**Figure S10** LOESS Analysis of Models’ Performance Following Age Distribution Comparison

**6. TRIPOD Checklist**

**Supplementary Table 8** TRIPOD Checklist (Prediction Model)

**7. Glossary of TCM Specific Terms Used**

**Supplementary Table 9** Cross-Reference of Synonym Terms in TCM with Definitions and Explanations

**8. Existing Research and Further Discussion on Predictors in the Final Models**

**9. Reference**

**1. Modeling Sample Size Calculation**

This study employed traditional Logistic regression and five machine learning algorithms for modeling. The sample size was calculated based on the Events per Predictor (EPP) rule. The EPP rule is commonly applied to traditional Logistic regression models to ensure model robustness and avoid overfitting. Although machine learning algorithms can learn features more deeply and require a smaller sample size, we still adopted this stricter standard to ensure the robustness of the models (Each predictor requires at least 15 events). In the modeling of the α/β model, even the traditional Logistic regression model, which performed worse than the final machine learning model, achieved an AUC value above 0.75 both in internal/external validation. This result indicates that the variables involved in this clinical problem do have objective associations. The model is constructed based on actual clinical data and the intrinsic relationships between variables, rather than being built for the sake of modeling. For the α model, which includes 7 clinical variables, there should be more than 105 = (15×7) pneumonia patients. For the β model, which introduces an additional 4 clinical variables, there should be more than 165 = (15×(7+4)) pneumonia patients. Considering all predictors and interaction terms used in the α/β models, there should be more than 285 = (15×(7+6+4+2)) pneumonia patients. Since the training cohort includes 362 pneumonia patients, the sample size requirement is met for modeling.

Although our study has an adequate sample size for the development of the predictive model, we must acknowledge the limitation of being a single-center study. Considering that BHTCM is a large Grade A Tertiary hospital located in the central urban area of Beijing, we believe that this institution, even as a single center, sufficiently represents the patient population in Beijing. The scale of the hospital also ensures an adequate number of emergency department visits, thereby supporting the sample size of this study. Although our model itself is trans-epidemic in nature, and it can be assumed that the two patient populations do not overlap temporally or spatially, the single-center design inevitably introduces limitations. Therefore, in subsequent research focused on the practical deployment, refinement, and updating of the model, we plan to collaborate with more regional hospitals outside Beijing to conduct large-scale, multi-center cohort studies.

**2. R Packages Used in Work**

*glmnet, ggplot2, corrplot, gridExtra, caret, pROC, xgboost, ada, ROCR, shiny, shapviz, car,* *nortest, DALEX, reshape2, klaR, gbm, readxl, dcurves, catboost, dplyr, compareGroups, forestplot, boot, mclust, rms, patchwork, openxlsx, poLCA, PredictABEL* packages are used in our work with RStudio. The modeling of the five algorithms—traditional Logistic regression, Logisticnet, Randomforest, XGBoost, and AdaBoost—is based on the *caret* package and other supplementary packages. The modeling of the CatBoost algorithm is based on the *CatBoost* package. Main code contents and directly analyzable datasets can be found in the supplementary files.​

**3. External Validation Cohort’s Clinical Characteristics**

After completing the model construction, we further compared the clinical characteristics between pneumonia patients (N=24) and non-pneumonia patients (N=186) in the external validation cohort (**Supplementary Table 1**). It is worth noting that this external validation cohort did not screen for disease diagnosis but included all patients who visited the fever clinic. Therefore, it more closely reflects the real-world clinical environment, differentiating not only patients with pneumonia-like symptoms but also covering all patients who visited the fever clinic.

In the real-world clinical setting, medical records in emergency and fever clinics often have missing data. Although we randomly selected 300 individuals from over 2,700 visits over six months, 90 cases were excluded from the final external validation cohort due to not meeting the inclusion and exclusion criteria. This situation may be related to the purpose of the patients' visits. Some patients visited the clinic merely to obtain and purchase medications as required by regulations, rather than for actual treatment. As a result, clinicians did not document their medical records properly. This phenomenon may lead to some cases not meeting the study's inclusion and exclusion criteria, thereby affecting the composition of the final cohort.

When comparing the clinical characteristics of the 210 patients ultimately included, we found that only age, cough, and CRP showed significant differences between groups (all P<0.05). Compared to the 7 clinical variables that previously showed significant differences in the internal training cohort, variables that previously had high OR, such as altered mental status and dyspnea, as well as Tmax, days, and pharyngeal discomfort, which are important in the α model, did not show significant differences in this comparison. However, after further examining interaction effects, we found that the interaction terms between age and Tmax, and between age and days of illness, were significantly different between groups (all P<0.05).

Despite the significant differences in clinical characteristics between groups in the internal training and external validation cohorts, the α model demonstrated stable AUC values in both cohorts (both AUC=0.80), which further confirms the robustness of our model. In the modeling of both the α and β models, we utilized the CatBoost algorithm, which exhibited the smallest changes in AUC values during internal and external validation. This algorithm not only outperformed other modeling methods but also demonstrated greater stability. Additionally, it showed significant advantages in IDI analysis (**Figure S1**). After upgrading to the β model, the AUC value in the internal validation significantly increased (DeLong Test P<0.001), while the increase in the external validation was not significant (DeLong Test P=0.748) (**Figure S2**). This discrepancy may be due to the differences in laboratory test indicators between the two cohorts. This finding suggests that in future modeling, we need more data from different epidemic environments to further optimize the model's performance.

Although the AUC remained stable, the ability of both models to identify negative classes was compromised during external validation (primarily due to a decline in the specificity of the α model). Meanwhile, the β model demonstrated significantly more stable performance than the α model after incorporating objective laboratory indicators. From a research design perspective, the loss of external generalizability may stem from differences in epidemiological contexts and cohort recruitment protocols between the internal and external validation cohorts. To clarify the models' limitations, we therefore conducted comparisons between the internal and external validation cohorts: first, an overall comparison between the two cohorts (**Supplementary Table 2**); followed by subgroup analyses comparing pneumonia patients (**Supplementary Table 3**) and non-pneumonia patients **(Supplementary Table 4)** between the two cohorts. Considering that the external validation cohort comprised fever patients in stable COVID-19 transmission settings, COVID-19 patients were compared with Non-COVID-19 patients in the external validation cohort (**Supplementary Table 5**), as well as with the overall internal validation cohort (**Supplementary Table 6**). Similarly, non-COVID-19 patients in the external validation cohort were compared with all patients in the internal validation cohort (**Supplementary Table 7**).

We observed that in the comparison of demographic characteristics between the internal and external validation cohorts, symptoms such as cough and pharyngeal discomfort, as well as the laboratory index NLR, all had very small P-values. Subsequent comparisons between the two groups of pneumonia and non-pneumonia patients in the subgroup analysis revealed that significant differences in these three clinical features persisted. Further comparisons were made to assess the impact of including COVID-19 patients on population differences. It was found that when compared with non-COVID-19 patients in the external validation cohort, differences in these three clinical features were not significant. However, when compared with the entire internal validation cohort, these differences re-emerged as significant. After excluding COVID-19 patients, the remaining external validation cohort still exhibited significant differences in these three clinical features when compared with the internal validation cohort.

Considering that the primary decline in the model's external generalizability is the reduced ability to identify negative cases of non-pneumonia patients, these differences in clinical features may be important mediating factors (all of which served as model predictors). In light of the changing epidemiological context, COVID-19 patients with the Omicron variant may experience more coughing, pharyngeal discomfort, and a decrease in NLR compared to patients in the previous epidemiological environment. The vast majority of these COVID-19 patients are non-pneumonia patients, which aligns with the reduced pathogenicity of the virus during the stable transmission period following the mutation of the strain. However, this also impacts the model's ability to distinguish negative cases. Other febrile patients in the new epidemiological environment still show differences in these three clinical features compared to patients in the old epidemiological environment. Part of this can be explained by the fact that the external validation cohort did not undergo the same inclusion and exclusion criteria (for example, patients with acute gastroenteritis or urinary tract infections). The other part should consider changes in the clinical manifestations caused by other respiratory pathogens (mainly influenza A and B viruses).

**Supplementary Table 1** Comparison of Characteristics between Pneumonia and Non-Pneumonia in the External Validation Cohort

| Characteristic | | Non-pneumonia | Pneumonia | Odds Ratio 95%CI | P value |
| --- | --- | --- | --- | --- | --- |
|  |  | (N=186) | (N=24) |  |  |
| COVID-19 | No=0 | 161 (86.6%) | 23 (95.8%) | 0.28 [0.02-1.43] | 0.323 |
|  | Yes=1 | 25 (13.4%) | 1 (4.2%) |  |  |
| Age(y) | | 40.1 (16.4) | 55.0 (19.7) | 1.04 [1.02-1.07] | 0.001^**^ |
| Days(d) | | 2.23 (2.21) | 4.58 (6.28) | 1.17 [1.04-1.32] | 0.081 |
| Tmax(℃) | | 38.3 (0.69) | 38.5 (0.88) | 1.62 [0.90-2.91] | 0.190 |
| Pharyngeal discomfort | No=0 | 65 (34.9%) | 9 (37.5%) | 0.89 [0.37-2.25] | 0.984 |
|  | Yes=1 | 121 (65.1%) | 15 (62.5%) |  |  |
| Cough | No=0 | 77 (41.4%) | 2 (8.33%) | 7.23 [2.03-49.9] | 0.003^**^ |
|  | Yes=1 | 109 (58.6%) | 22 (91.7%) |  |  |
| Dyspnea | No=0 | 184 (98.9%) | 22 (91.7%) | 8.20 [0.82-82.1] | 0.065 |
|  | Yes=1 | 2 (1.08%) | 2 (8.33%) |  |  |
| Altered mental status | No=0 | 183 (98.4%) | 22 (91.7%) | 5.59 [0.62-38.7] | 0.101 |
|  | Yes=1 | 3 (1.61%) | 2 (8.33%) |  |  |
| Age*tmax | | 1536 (626) | 2121 (779) | 1.00 [1.00-1.00] | 0.001^**^ |
| Age*days | | 90.1 (106) | 258 (338) | 1.00 [1.00-1.01] | 0.024^*^ |
| Age*altered mental status | | 0.96 (8.11) | 8.00 (27.1) | 1.03 [1.00-1.05] | 0.219 |
| Age*pharyngeal discomfort | | 25.2 (21.9) | 31.5 (28.1) | 1.01 [0.99-1.03] | 0.306 |
| Age*cough | | 22.7 (22.5) | 47.2 (21.4) | 1.05 [1.02-1.07] | <0.001^***^ |
| Age*dyspnea | | 0.71 (7.30) | 7.29 (25.0) | 1.03 [1.00-1.06] | 0.212 |
| NLR | | 5.56 (4.24) | 7.76 (11.7) | 1.05 [0.99-1.11] | 0.368 |
| CRP | | 22.2 (30.9) | 45.2 (45.6) | 1.01 [1.00-1.02] | 0.024^*^ |
| PLT | | 221 (64.6) | 226 (70.7) | 1.00 [0.99-1.01] | 0.729 |
| CRP/PLT | | 0.12 (0.19) | 0.20 (0.21) | 5.69 [1.04-31.2] | 0.056 |

Mean(SD) ; n(%), *P<0.05, **P<0.01, ***P<0.001.

**Supplementary Table 2** Comparison of Characteristics between the Internal/External Validation Cohorts

| Characteristic | | Internal validation cohort | External validation cohort | P value |
| --- | --- | --- | --- | --- |
|  |  | (N=1781) | (N=210) |  |
| Age(y) | | 43.84 (20.55) | 41.83 (17.39) | 0.782 |
| Days(d) | | 2.77 (4.15) | 2.50 (3.04) | 0.86 |
| Tmax(℃) | | 38.19 (0.66) | 38.30 (0.71) | 0.01^*^ |
| Pharyngeal discomfort | No=0 | 1370 (76.9%) | 74 (35.2%) | <0.001^***^ |
|  | Yes=1 | 411 (23.1%) | 136 (64.8%) |  |
| Cough | No=0 | 1531 (86.0%) | 79 (37.6%) | <0.001^***^ |
|  | Yes=1 | 250 (14.0%) | 131 (62.4%) |  |
| Dyspnea | No=0 | 1745 (98.0%) | 206 (98.1%) | 1 |
|  | Yes=1 | 36 (2.0%) | 4 (1.9%) |  |
| Altered mental status | No=0 | 1733 (97.3%) | 205 (97.6%) | 0.967 |
|  | Yes=1 | 48 (2.7%) | 5 (2.4%) |  |
| Age*tmax | | 1675.5 (789.21) | 1602.4 (669.27) | 0.861 |
| Age*days | | 128.11 (226.50) | 109.2 (159.43) | 0.683 |
| Age*altered mental status | | 2.21 (13.43) | 1.77 (12.01) | 0.793 |
| Age*pharyngeal discomfort | | 8.11 (16.17) | 25.96 (22.68) | <0.001^***^ |
| Age*cough | | 6.85 (18.73) | 25.51 (23.64) | <0.001^***^ |
| Age*dyspnea | | 1.41 (10.16) | 1.46 (10.97) | 0.919 |
| NLR | | 7.54 (7.80) | 5.81 (5.60) | <0.001^***^ |
| CRP | | 31.30 (45.48) | 24.81 (33.58) | 0.144 |
| PLT | | 218.57 (66.13) | 221.21 (65.20) | 0.472 |
| CRP/PLT | | 0.16 (0.26) | 0.13 (0.19) | 0.129 |

Mean(SD) ; n(%), *P<0.05, **P<0.01, ***P<0.001.

**Supplementary Table 3** Comparison of Characteristics between Pneumonia Patients in the Internal/External Validation Cohorts

| Characteristic | | Internal pneumonia | External pneumonia | P value |
| --- | --- | --- | --- | --- |
|  |  | (N=362) | (N=24) |  |
| Age(y) | | 62.22 (22.17) | 54.96 (19.71) | 0.083 |
| Days(d) | | 3.50 (4.49) | 4.58 (6.28) | 0.259 |
| Tmax(℃) | | 38.32 (0.71) | 38.52 (0.88) | 0.118 |
| Pharyngeal discomfort | No=0 | 330 (91.2%) | 9 (37.5%) | <0.001^***^ |
|  | Yes=1 | 32 (8.8%) | 15 (62.5%) |  |
| Cough | No=0 | 290 (80.1%) | 2 (8.3%) | <0.001^***^ |
|  | Yes=1 | 72 (19.9%) | 22 (91.7%) |  |
| Dyspnea | No=0 | 343 (94.8%) | 22 (91.7%) | 0.381 |
|  | Yes=1 | 19 (5.2%) | 2 (8.3%) |  |
| Altered mental status | No=0 | 325 (89.8%) | 22 (91.7%) | 1 |
|  | Yes=1 | 37 (10.2%) | 2 (8.3%) |  |
| Age*tmax | | 2385.29 (850.64) | 2120.62 (778.76) | 0.083 |
| Age*days | | 226.16 (330.41) | 257.54 (338.07) | 0.614 |
| Age*altered mental status | | 8.55 (25.55) | 8.00 (27.13) | 0.851 |
| Age*pharyngeal discomfort | | 4.25 (14.44) | 31.46 (28.09) | <0.001^***^ |
| Age*cough | | 12.60 (26.77) | 47.21 (21.41) | <0.001^***^ |
| Age*dyspnea | | 4.14 (17.79) | 7.29 (24.98) | 0.500 |
| NLR | | 11.90 (12.72) | 7.76 (11.66) | 0.002^**^ |
| CRP | | 66.45 (68.81) | 45.19 (45.61) | 0.307 |
| PLT | | 212.99 (78.15) | 225.92 (70.73) | 0.399 |
| CRP/PLT | | 0.36 (0.42) | 0.20 (0.21) | 0.231 |

Mean(SD) ; n(%), *P<0.05, **P<0.01, ***P<0.001.

**Supplementary Table 4** Comparison of Characteristics between Non-Pneumonia Patients in the Internal/External Validation Cohorts

| Characteristic | | Internal non-pneumonia | External non-pneumonia | P value |
| --- | --- | --- | --- | --- |
|  |  | (N=1419) | (N=186) |  |
| Age(y) | | 39.15 (17.22) | 40.13 (16.38) | 0.137 |
| Days(d) | | 2.58 (4.05) | 2.23 (2.21) | 0.854 |
| Tmax(℃) | | 38.15 (0.64) | 38.28 (0.69) | 0.012^*^ |
| Pharyngeal discomfort | No=0 | 1040 (73.3%) | 65 (34.9%) | <0.001^***^ |
|  | Yes=1 | 379 (26.7%) | 121 (65.1%) |  |
| Cough | No=0 | 1241 (87.5%) | 77 (41.4%) | <0.001^***^ |
|  | Yes=1 | 178 (12.5%) | 109 (58.6%) |  |
| Dyspnea | No=0 | 1402 (98.8%) | 184 (98.9%) | 1 |
|  | Yes=1 | 17 (1.2%) | 2 (1.1%) |  |
| Altered mental status | No=0 | 1408 (99.2%) | 183 (98.4%) | 0.215 |
|  | Yes=1 | 11 (0.8%) | 3 (1.6%) |  |
| Age*tmax | | 1494.36 (660.45) | 1535.58 (625.54) | 0.109 |
| Age*days | | 103.09 (183.10) | 90.10 (106.23) | 0.579 |
| Age*altered mental status | | 0.59 (6.87) | 0.96 (8.11) | 0.250 |
| Age*pharyngeal discomfort | | 9.10 (16.44) | 25.25 (21.88) | <0.001^***^ |
| Age*cough | | 5.38 (15.73) | 22.71 (22.48) | <0.001^***^ |
| Age*dyspnea | | 0.71 (6.83) | 0.71 (7.30) | 0.887 |
| NLR | | 6.43 (5.38) | 5.56 (4.24) | 0.013^*^ |
| CRP | | 22.33 (31.54) | 22.18 (30.90) | 0.654 |
| PLT | | 220.00 (62.65) | 220.60 (64.63) | 0.772 |
| CRP/PLT | | 0.11 (0.17) | 0.12 (0.19) | 0.631 |

Mean(SD) ; n(%), *P<0.05, **P<0.01, ***P<0.001.

**Supplementary Table 5** Comparison of Characteristics between COVID-19 and Non-COVID-19 in the External Validation Cohort

| Characteristic | | External non-COVID-19 | External COVID-19 | P value |
| --- | --- | --- | --- | --- |
|  |  | (N=184) | (N=26) |  |
| Pneumonia | No=0 | 161 (87.5%) | 25 (96.2%) | 0.323 |
|  | Yes=1 | 23 (12.5%) | 1 (3.8%) |  |
| Age(y) | | 40.89 (16.97) | 48.50 (19.19) | 0.050 |
| Days(d) | | 2.62 (3.21) | 1.65 (0.80) | 0.092 |
| Tmax(℃) | | 38.33 (0.72) | 38.12 (0.66) | 0.159 |
| Pharyngeal discomfort | No=0 | 66 (35.9%) | 8 (30.8%) | 0.772 |
|  | Yes=1 | 118 (64.1%) | 18 (69.2%) |  |
| Cough | No=0 | 70 (38.0%) | 9 (34.6%) | 0.903 |
|  | Yes=1 | 114 (62.0%) | 17 (65.4%) |  |
| Dyspnea | No=0 | 180 (97.8%) | 26 (100.0%) | 1 |
|  | Yes=1 | 4 (2.2%) | 0 (0.0%) |  |
| Altered mental status | No=0 | 180 (97.8%) | 25 (96.2%) | 0.487 |
|  | Yes=1 | 4 (2.2%) | 1 (3.8%) |  |
| Age*tmax | | 1567.50 (654.44) | 1849.73 (732.74) | 0.052 |
| Age*days | | 112.43 (168.43) | 86.62 (65.07) | 0.793 |
| Age*altered mental status | | 1.72 (12.23) | 2.08 (10.59) | 0.615 |
| Age*pharyngeal discomfort | | 25.47 (22.49) | 29.42 (24.15) | 0.398 |
| Age*cough | | 24.80 (22.99) | 30.54 (27.77) | 0.408 |
| Age*dyspnea | | 1.67 (11.71) | 0.00 (0.00) | 0.453 |
| NLR | | 6.02 (5.80) | 4.33 (3.60) | 0.077 |
| CRP | | 26.45 (35.02) | 13.21 (16.98) | 0.033^*^ |
| PLT | | 224.92 (65.40) | 194.96 (58.46) | 0.014^*^ |
| CRP/PLT | | 0.13 (0.20) | 0.07 (0.09) | 0.116 |

Mean(SD) ; n(%), *P<0.05, **P<0.01, ***P<0.001.

**Supplementary Table 6** Comparison of Characteristics between COVID-19 and the Internal Validation Cohort (surely non-COVID-19)

| Characteristic | | Internal non-COVID-19 | External COVID-19 | P value |
| --- | --- | --- | --- | --- |
|  |  | (N=1781) | (N=26) |  |
| Pneumonia | No=0 | 1419 (79.7%) | 25 (96.2%) | 0.045^*^ |
|  | Yes=1 | 362 (20.3%) | 1 (3.8%) |  |
| Age(y) | | 43.84 (20.55) | 48.50 (19.19) | 0.143 |
| Days(d) | | 2.77 (4.15) | 1.65 (0.80) | 0.134 |
| Tmax(℃) | | 38.19 (0.66) | 38.12 (0.66) | 0.768 |
| Pharyngeal discomfort | No=0 | 1370 (76.9%) | 8 (30.8%) | <0.001^***^ |
|  | Yes=1 | 411 (23.1%) | 18 (69.2%) |  |
| Cough | No=0 | 1531 (86.0%) | 9 (34.6%) | <0.001^***^ |
|  | Yes=1 | 250 (14.0%) | 17 (65.4%) |  |
| Dyspnea | No=0 | 1745 (98.0%) | 26 (100.0%) | 1 |
|  | Yes=1 | 36 (2.0%) | 0 (0.0%) |  |
| Altered mental status | No=0 | 1733 (97.3%) | 25 (96.2%) | 0.513 |
|  | Yes=1 | 48 (2.7%) | 1 (3.8%) |  |
| Age*tmax | | 1675.45 (789.21) | 1849.73 (732.74) | 0.153 |
| Age*days | | 128.11 (226.50) | 86.62 (65.07) | 0.684 |
| Age*altered mental status | | 2.21 (13.43) | 2.08 (10.59) | 0.744 |
| Age*pharyngeal discomfort | | 8.11 (16.17) | 29.42 (24.15) | <0.001^***^ |
| Age*cough | | 6.85 (18.73) | 30.54 (27.77) | <0.001^***^ |
| Age*dyspnea | | 1.41 (10.16) | 0.00 (0.00) | 0.465 |
| NLR | | 7.54 (7.80) | 4.33 (3.60) | <0.001^***^ |
| CRP | | 31.30 (45.48) | 13.21 (16.98) | 0.011^*^ |
| PLT | | 218.57 (66.13) | 194.96 (58.46) | 0.045^*^ |
| CRP/PLT | | 0.16 (0.26) | 0.07 (0.09) | 0.038^*^ |

Mean(SD) ; n(%), *P<0.05, **P<0.01, ***P<0.001.

**Supplementary Table 7** Comparison of Characteristics between non-COVID-19 and the Internal Validation Cohort

| Characteristic | | Internal non-COVID-19 | External non-COVID-19 | P value |
| --- | --- | --- | --- | --- |
|  |  | (N=1781) | (N=184) |  |
| Pneumonia | No=0 | 1419 (79.7%) | 161 (87.5%) | 0.014^*^ |
|  | Yes=1 | 362 (20.3%) | 23 (12.5%) |  |
| Age(y) | | 43.84 (20.55) | 40.89 (16.97) | 0.409 |
| Days(d) | | 2.77 (4.15) | 2.62 (3.21) | 0.727 |
| Tmax(℃) | | 38.19 (0.66) | 38.33 (0.72) | 0.004^**^ |
| Pharyngeal discomfort | No=0 | 1370 (76.9%) | 66 (35.9%) | <0.001^***^ |
|  | Yes=1 | 411 (23.1%) | 118 (64.1%) |  |
| Cough | No=0 | 1531 (86.0%) | 70 (38.0%) | <0.001^***^ |
|  | Yes=1 | 250 (14.0%) | 114 (62.0%) |  |
| Dyspnea | No=0 | 1745 (98.0%) | 180 (97.8%) | 0.785 |
|  | Yes=1 | 36 (2.0%) | 4 (2.2%) |  |
| Altered mental status | No=0 | 1733 (97.3%) | 180 (97.8%) | 1 |
|  | Yes=1 | 48 (2.7%) | 4 (2.2%) |  |
| Age*tmax | | 1675.45 (789.21) | 1567.50 (654.44) | 0.482 |
| Age*days | | 128.11 (226.50) | 112.43 (168.43) | 0.770 |
| Age*altered mental status | | 2.21 (13.43) | 1.72 (12.23) | 0.687 |
| Age*pharyngeal discomfort | | 8.11 (16.17) | 25.47 (22.49) | <0.001^***^ |
| Age*cough | | 6.85 (18.73) | 24.80 (22.99) | <0.001^***^ |
| Age*dyspnea | | 1.41 (10.16) | 1.67 (11.71) | 0.880 |
| NLR | | 7.54 (7.80) | 6.02 (5.80) | 0.001^**^ |
| CRP | | 31.30 (45.48) | 26.45 (35.02) | 0.512 |
| PLT | | 218.57 (66.13) | 224.92 (65.40) | 0.135 |
| CRP/PLT | | 0.16 (0.26) | 0.13 (0.20) | 0.375 |

Mean(SD) ; n(%), *P<0.05, **P<0.01, ***P<0.001.


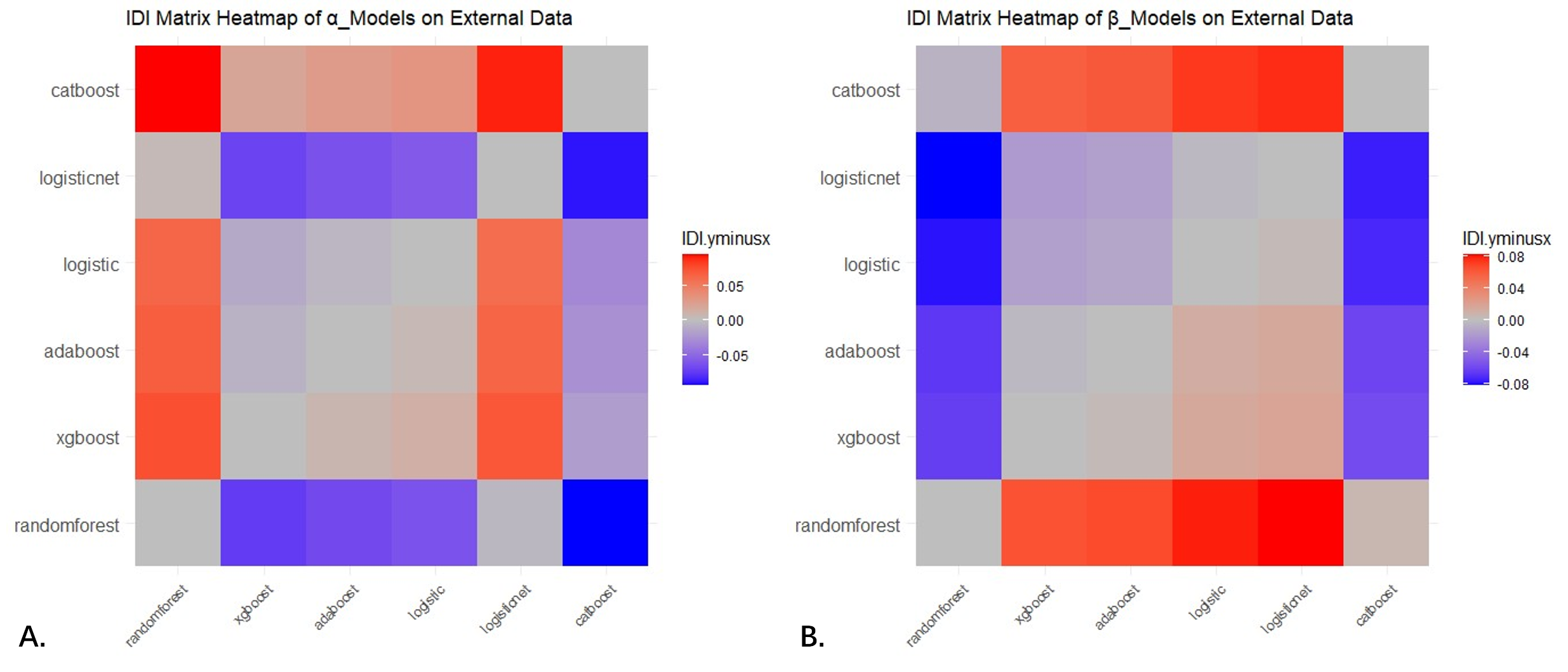


**Figure S1** The Heatmap of IDI Analysis for the α/β Models in External Validation (**A.** α_model’s IDI analysis; **B.** β_model’s IDI analysis)


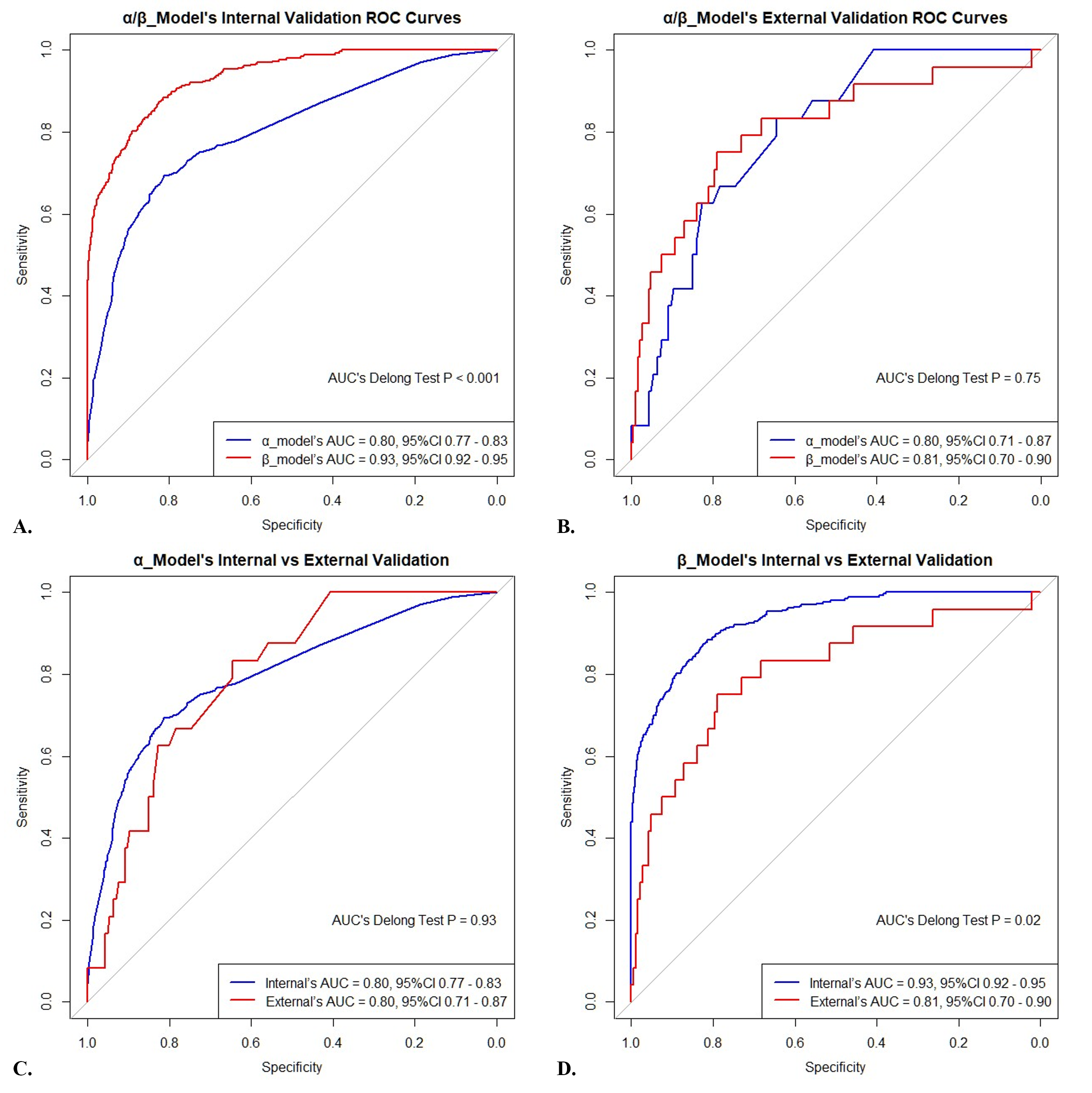


**Figure S2** Comparison of the ROC Curves for the Final α/β_Models (**A.** internal validation; **B.** external validation; **C**. α_Model; **D**. β_Model)

**4. Discussion of CAP Diagnostic Criteria**

The existing CAP diagnostic criteria provide vague descriptions of clinical symptoms, requiring only a few symptoms related to pulmonary infection. Unless the patients condition is severe, cough and sputum production alone are not sufficient to clinically distinguish pneumonia from patients with similar symptoms (such as upper respiratory infections). However, our model incorporates a wide range of clinical symptoms and further differentiates various clinical subtypes of CAP based on these symptoms. As a further exploration based on existing guidelines, we conducted unsupervised automatic latent class analysis in 1,781 patients from the internal training and validation cohorts (**Figure S3**). The results showed that, in addition to the obvious symptoms of altered mental status and dyspnea, pneumonia patients were distinguished from another class by the combination of clinical manifestations including fatigue, pharyngeal discomfort, head&body pain, nasal&congestion, and abdominal pain. We further constructed a heatmap of the correlations between all clinical feature variables among these patients. Many clinical symptoms, as well as laboratory tests (such as WBC), showed clear correlations with pneumonia (**Figure S4**). Thus, we suggest that in the future updates of CAP guidelines, more research on the diagnostics of clinical symptoms should be conducted, not only to identify CAP patients but also to differentiate the clinical subtypes of CAP patients.


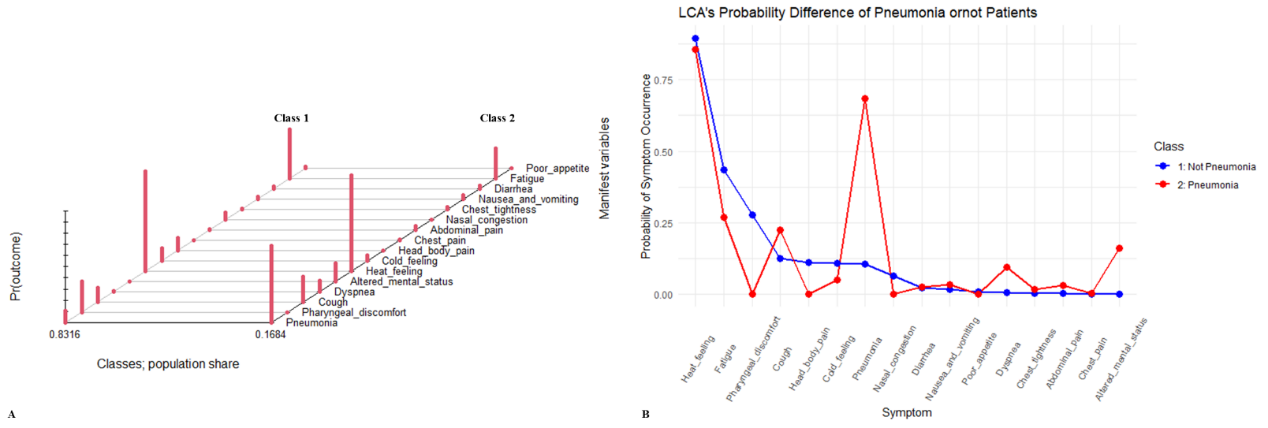


**Figure S3** Series of Latent Class Analysis Charts for Automatic Subtype Classification of internal Training Cohort **(A.** The LCA plot of two latent classes demonstrating the distinction of pneumonia or not; **B.** The plot comparing LCA's probability difference of pneumonia ornot patients based on two lacent classes)


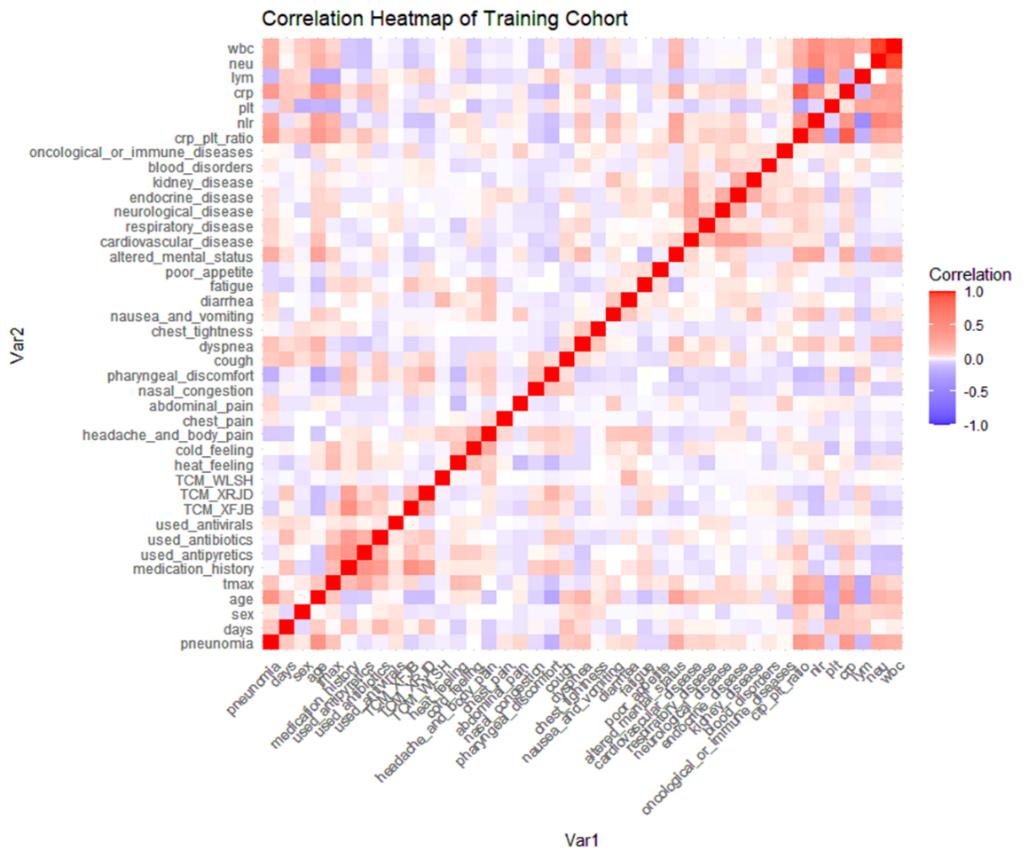


**Figure S4** The Correlation Heatmap among Clinical Variables in the Training Cohort

**5. Supplementary Calibration Figures and Models’ Performance across Subgroups**

We have supplemented the details of the calibration curve-related figures in the main text here, including separate calibration curve plots for each algorithm compared during the internal and external validation of the two models, along with the linear fit slopes and intercepts of their respective curves. These results provide references for our final models and the selection strategy of specific algorithms (**Figure S5 to S8**). As described in the main text, the Randomforest algorithm exhibited overfitting during the internal validation of the β-model, rendering its calibration curve ineffective for meaningful interpretation. Among the remaining comparisons, the CatBoost algorithm demonstrated superior performance in both the α and β models. Furthermore, the β-model overall showed better and more stable performance in calibration curves compared to the α-model. This highlights the effectiveness of the staged modeling strategy, where incorporating laboratory test parameters as a supplement to clinical manifestations enhanced the model's screening capability.

We extended our analysis by comparing ROC curves across subgroups (e.g., sex) to evaluate the model's relationship with previously unexamined clinical variables, serving as a supplementary analysis to our primary findings. The performance of both models remained robust even when assessed on subgroups defined by these new variables (**Figure S9**). The ROC curves exhibit increased jaggedness during external validation, which is attributable to the substantial reduction in sample size resulting from artificial subgroup partitioning. In contrast, the curves remain smooth in the internal cohort with a larger overall sample size. Even when assessed exclusively within the internal cohort, both models demonstrate no significant performance bias across gender-based subgroups. Given that gender was not incorporated as a predictor in the model training process, the consistent performance observed between male and female subgroups underscores the stability of the models' predictive capability independent of sex-related variables.

We are interested in the characteristics of patients for which the models are more applicable. However, when age is treated as a continuous variable, artificially defining cutpoints to create subgroups can lead to significant information loss, reduced statistical power, and residual confounding[1]. Therefore, we employed a LOESS (Locally Estimated Scatterplot Smoothing) fitting method from nonparametric regression. This approach divides the patient population into sliding windows based on age, with a window width set to 10 years, and then calculates the model's AUC value within each window. While this method essentially remains a form of subgroup analysis, it is more objective compared to manually defining cutpoints.

Based on the results, the AUC of the α_model showed some decrease during internal validation, whereas the AUC of the β_model improved (**Figure S10**). This is likely because the α_model objectively introduced age dependency through age interaction terms, while the β_model incorporated improvements via a phased modeling strategy that objectively included laboratory indicators. However, during external validation, which was entirely distinct from the training environment, the AUC values of both models performed ideally or even better across most age intervals. Overall, both final models demonstrated the best screening performance in the 45-65 age range, which may be related to the underrepresentation of the oldest old adults in the training data. Considering that subgroup analysis of continuous variables inherently reduces statistical power with a big cut of sample size in each subgroups, intentionally adding more samples from the oldest old adults for training in future model updates and improvements will be a key focus.


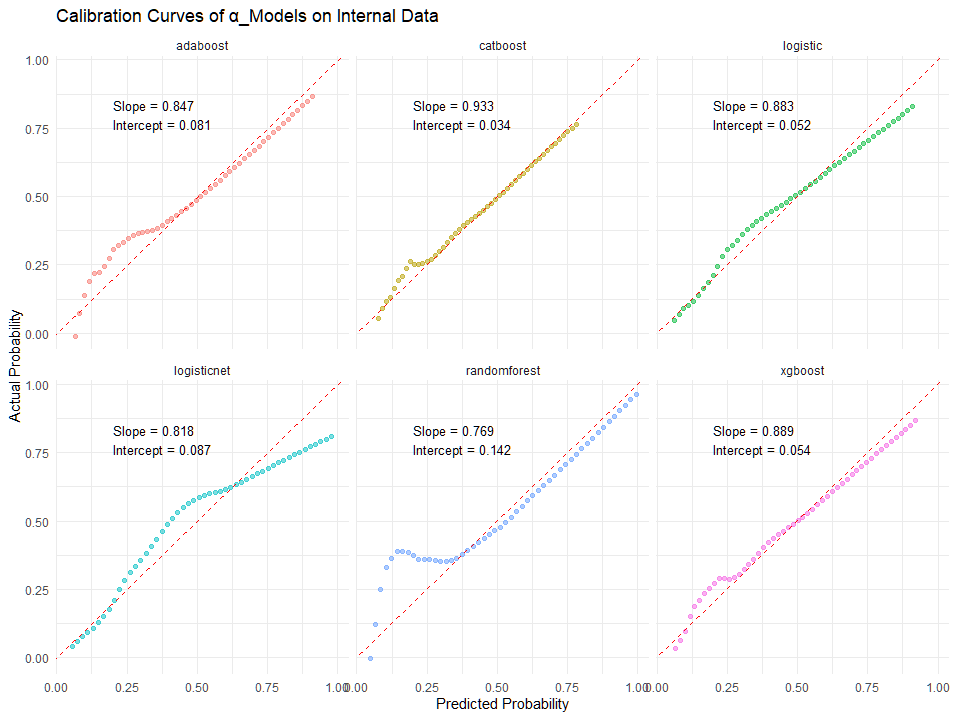


**Figure S5** Calibration Curves of α_Models on Internal Dataset between 6 Algorithms


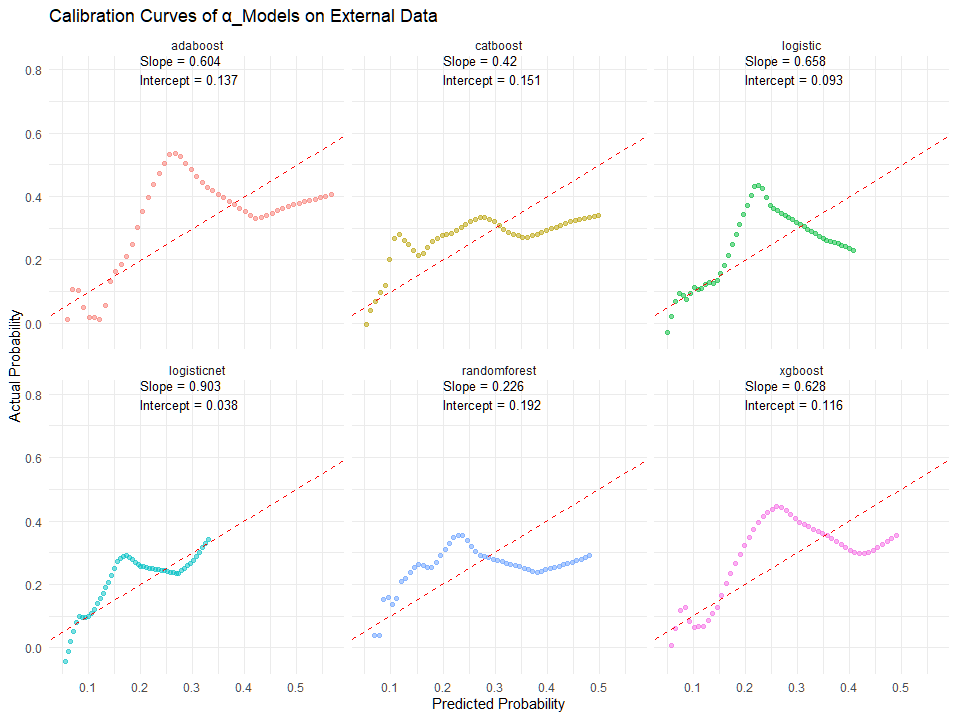


**Figure S6** Calibration Curves of α_Models on External Dataset between 6 Algorithms


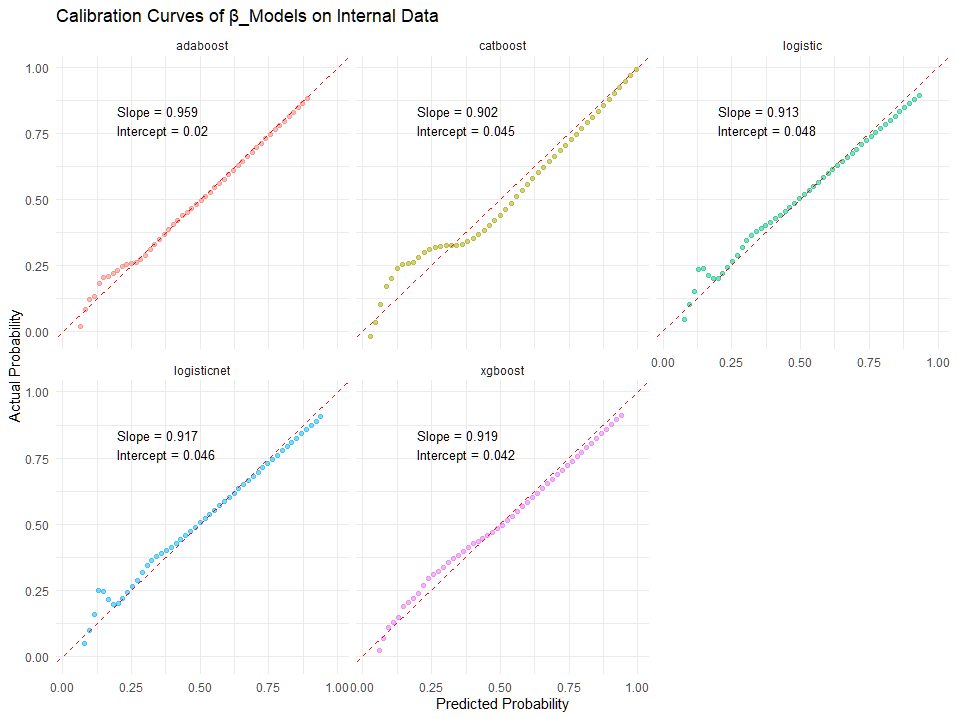


**Figure S7** Calibration Curves of β_Models on Internal Dataset between 5 Algorithms


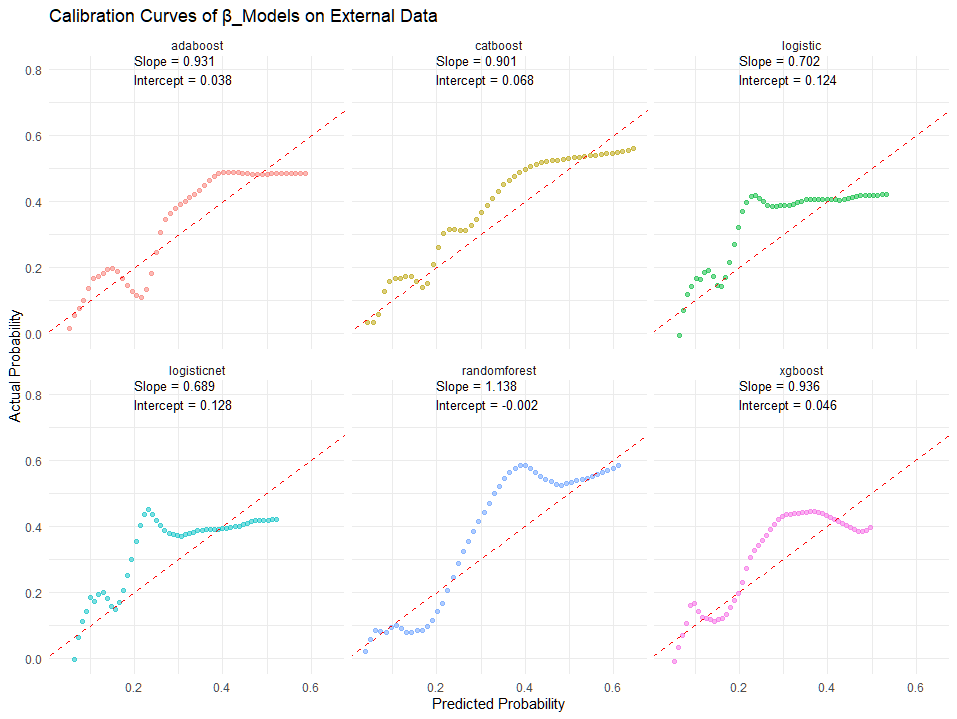


**Figure S8** Calibration Curves of β_Models on External Dataset between 6 Algorithms


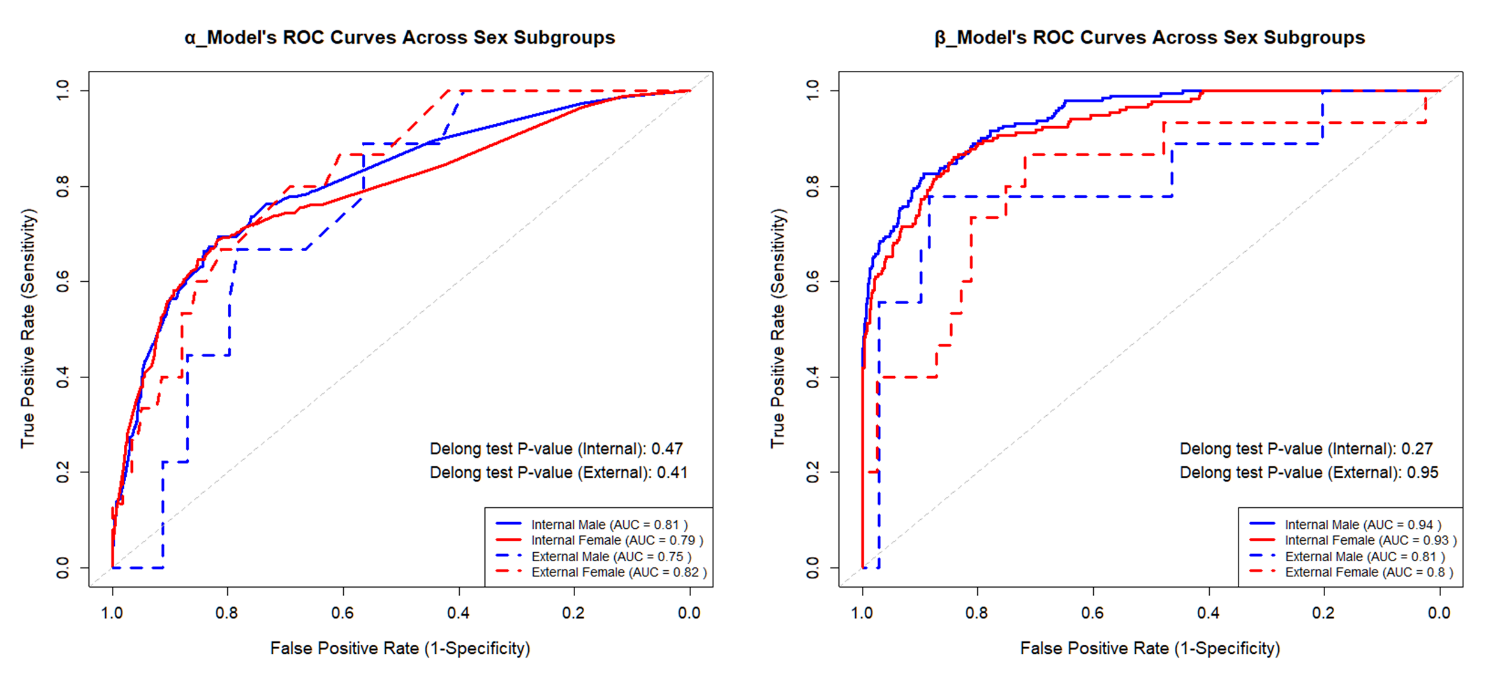


**Figure S9** Final Models’ ROC Curves Across Sex Subgroups


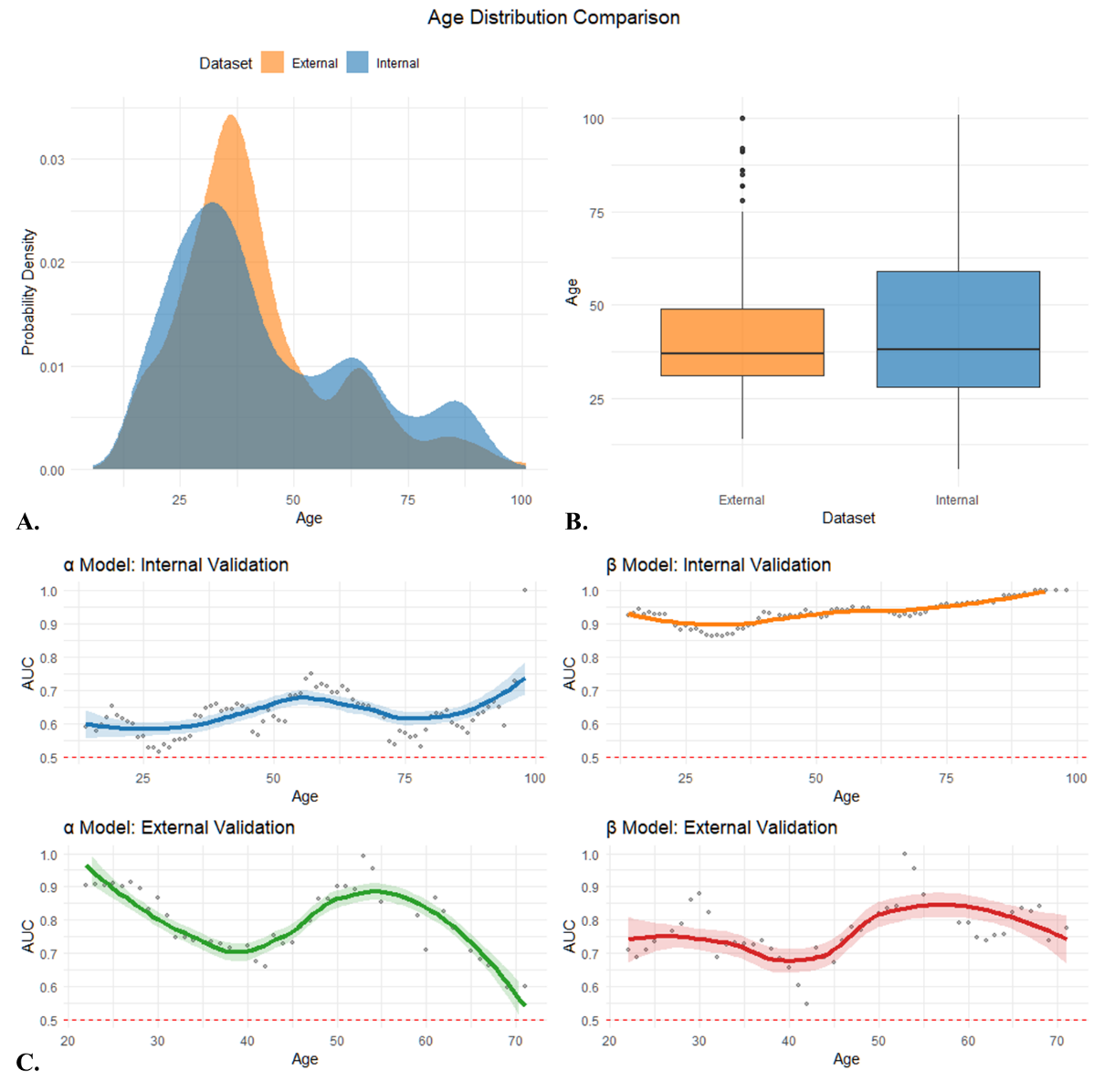


**Figure S10** LOESS Analysis of Models’ Performance Following Age Distribution Comparison

**
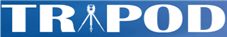
6. TRIPOD Checklist**

**Supplementary Table 8** TRIPOD Checklist (Prediction Model)

| **Section/Topic** | **Item** | **Checklist Item** | **Page** |
| --- | --- | --- | --- |
| **Title＆abstract** |  |  |  |
| Title | 1 | Identify the study as developing and/or validating a multivariable prediction model, the target population, and the outcome to be predicted. | 1 |
| Abstract | 2 | Provide a summary of objectives, study design, setting, participants, sample size, predictors, outcome, statistical analysis, results, and conclusions. | 1 |
| **Introduction** |  |  |  |
| Background and objectives | 3a | Explain the medical context (including whether diagnostic or prognostic) and rationale for developing or validating the multivariable prediction model, including references to existing models. | 2 |
|  | 3b | Specify the objectives, including whether the study describes the development or validation of the model or both. | 2 |
| **Methods** |  |  |  |
| Source of data | 4a | Describe the study design or source of data (e.g., randomized trial, cohort, or registry data), separately for the development and validation data sets, if applicable. | 2-3 |
|  | 4b | Specify the key study dates, including start of accrual; end of accrual; and, if applicable, end of follow-up. | 2-3 |
| Participants | 5a | Specify key elements of the study setting (e.g., primary care, secondary care, general population) including number and location of centres. | 2-3 |
|  | 5b | Describe eligibility criteria for participants. | 2-3 |
|  | 5c | Give details of treatments received, if relevant. | / |
| Outcome | 6a | Clearly define the outcome that is predicted by the prediction model, including how and when assessed. | 2,4 |
|  | 6b | Report any actions to blind assessment of the outcome to be predicted. | 2,4 |
| Predictors | 7a | Clearly define all predictors used in developing or validating the multivariable prediction model, including how and when they were measured. | 2,4 |
|  | 7b | Report any actions to blind assessment of predictors for the outcome and other predictors. | 2,4 |
| Sample size | 8 | Explain how the study size was arrived at. | Supplement |
| Missing data | 9 | Describe how missing data were handled (e.g., complete-case analysis, single imputation, multiple imputation) with details of any imputation method. | 2-4 |
| Statistical analysis methods | 10a | Describe how predictors were handled in the analyses. | 3,4 |
|  | 10b | Specify type of model, all model-building procedures (including any predictor selection), and method for internal validation. | 3,4 |
|  | 10d | Specify all measures used to assess model performance and, if relevant, to compare multiple models. | 3,4 |
| Risk groups | 11 | Provide details on how risk groups were created, if done. | / |
| **Results** |  |  |  |
| Participants | 13a | Describe the flow of participants through the study, including the number of participants with and without the outcome and, if applicable, a summary of the follow-up time. A diagram may be helpful. | 4-6 |
|  | 13b | Describe the characteristics of the participants (basic demographics, clinical features, available predictors), including the number of participants with missing data for predictors and outcome. | 4-6 |
| Model development | 14a | Specify the number of participants and outcome events in each analysis. | 7-8 |
|  | 14b | If done, report the unadjusted association between each candidate predictor and outcome. | 7-8 |
| Model specification | 15a | Present the full prediction model to allow predictions for individuals (i.e., all regression coefficients, and model intercept or baseline survival at a given time point). | 9 |
|  | 15b | Explain how to the use the prediction model. | 8-9 |
| Model performance | 16 | Report performance measures (with CIs) for the prediction model. | 7-8,  Supplement |
| **Discussion** |  |  |  |
| Limitations | 18 | Discuss any limitations of the study (such as nonrepresentative sample, few events per predictor, missing data). | 9-10 |
| Interpretation | 19b | Give an overall interpretation of the results, considering objectives, limitations, and results from similar studies, and other relevant evidence. | 9-10 |
| Implications | 20 | Discuss the potential clinical use of the model and implications for future research. | 9-10 |
| **Other information** |  |  |  |
| Supplementary information | 21 | Provide information about the availability of supplementary resources, such as study protocol, Web calculator, and data sets. | Supplement |
| Funding | 22 | Give the source of funding and the role of the funders for the present study. | 4 |

**7. Glossary of TCM Specific Terms Used**

Traditional Chinese Medicine (TCM) plays a significant role in primary care settings across China. Existing clinical research on TCM provides evidence-based medical evidence supporting its efficacy, including herbal medicine treatments for severe pneumonia, COVID-19, influenza A, and other conditions[2][3][4][5]. Our team recently conducted a comprehensive review of the mechanisms underlying TCM treatments for pneumonia[6]. To facilitate academic exchange with peers in other countries and regions, we have added standardized definitions for TCM-specific terminology referenced in the text, ​in strict accordance with the WHO’s official TCM terminology translation standards​[7].

In this study, the classification of TCM subtypes was non-deliberate and automated. We merely employed an unsupervised learning algorithm—latent class analysis with four classes—to automatically cluster patients based on symptoms into distinct groups. However, it was evident that the first cluster represented severe CAP patients with impaired consciousness. Therefore, we consider this algorithm meaningful and proceeded to examine the remaining two categories, which precisely corresponded to the scope of TCM Cold syndrome and Heat syndrome subtypes. Thus, it can be considered that, as an supplementary investigation to the clinical diagnostic prediction model, the symptom-based clinical subtyping—particularly the TCM subtype classification—serves as a valuable addition to the original research. Now, both physicians and patients can not only understand the probability of pneumonia risk but also identify which TCM subtype of pneumonia is present. The standardized terminology provided below not only translates the specialized terms "Cold syndrome" and "Heat syndrome" but also includes supplementary descriptions. In the future, we will conduct more clinical and fundamental research related to TCM subtypes, particularly studies focusing on interventional treatments and prognostic outcomes. The diagnostic-related research involved in this paper has provided us with confidence, demonstrating that ancient medical experience remains valuable and worthy of research and refinement.

We maintain that even without considering these TCM concepts, this study can still serve as a medium for communication and a reference for practical clinical exploration with healthcare institutions in other regions on the objective aspects of CAP-related diseases, symptoms, and signs. Moreover, we welcome more critical discussions on the correlation between these objective manifestations and TCM concepts.

**Supplementary Table 9** Cross-Reference of Synonym Terms in TCM with Definitions and Explanations

| English term | Synonyms | English definition/description | Chinese Pinyin term |
| --- | --- | --- | --- |
| Cold syndrome | Cold pattern | Characterized by aversion to cold, cold intolerance, cold pain with preference for warmth, absence of thirst, thin, clear phlegm and nasal discharge, clear, profuse urine, loose stools and a pale complexion. The tongue is purple with a white coating. The pulse is tight or slow. Often results from excess of yin qi or cold attacking the interior. | hán zhèng |
| Heat syndrome | Heat pattern | Characterized by fever, a red face, thirst, constipation and yellow or red urine. Alternatively, malar flush, night sweats, feverish sensations in the palms, soles and chest, a dry, red tongue and a thready, rapid pulse may be present. It is sub-categorized into patterns of exterior heat, interior heat, deficiency heat and excess heat. Often results from contracting pathogenic heat or hyperactivity of yang due to yin deficiency. | rè zhèng |
| External Cold | Exterior cold pattern | Characterized by severe aversion to cold, mild fever, headache, and joint soreness/pain. The tongue coating is thin and white. The pulse is superficial and tight. Often occurs when wind cold attacks the surface of the body. | biǎo hán zhèng |
| Internal Heat | Interior heat pattern | Characterized by persistent high fever, red face and eyes, restlessness, thirst with a desire to drink cold water, constipation and scanty, dark yellow urine. In severe cases, unconsciousness and delirium may be present. The tongue is red with a dry, yellow coating. The pulse is deep and excessive. Often occurs when external pathogenic warm heat enters the interior. | lǐ rè zhèng |
| Superficial syndromes | Exterior pattern | Characterized by aversion to wind/cold, fever, headache, and body aches. The tongue coating is thin. The pulse is floating. These signs and symptoms are often seen in the early stage of externally contracted conditions that have a sudden onset, superficial location and a short duration. Often occurs when exogenous pathogenic factors affect the interstices, joints and head. | biǎo zhèng |
| Interior syndromes | Interior pattern | A group of patterns caused by entering of external pathogens into the interior, improper diet, over exhaustion, emotional disturbance or phlegm stasis, resulting in disorders and dysfunctions in qi, blood, yin, yang, zang–fu organs and meridian systems. | lǐ zhèng |
| Warming the Cold | Warming method | A method to warm the interior to eliminate pathogenic cold. | wēn fǎ |
|  | Warm yang and dissipate cold | A treatment method to warm and tonify yang qi and dissipate cold. It is indicated for cold retention due to yang deficiency. | wēn yáng sàn hán |
| Clearing the Heat | Clearing method | A method to clear heat, reduce fire, remove toxins and cool blood to eliminate pathogenic heat in the interior. | qīng fǎ |
|  | Clear heat and remove toxins | A treatment method to clear heat and reduce fire to remove toxins. It is indicated for toxic fire, migratory toxic fire or toxic fire entering the collaterals. | qīng rè jiě dú |

**8. Existing Research on Predictors in the Final Models**

In our final α/β model, there are 11 predictors: age, days, Tmax, pharyngeal discomfort, cough, dyspnea, altered mental status, NLR, and CRP/PLT. In the Discussion section, we have specifically analyzed the interaction between age and other variables, with detailed explanation of how the last two laboratory parameters may potentially influence the clinical course through pathophysiological processes. This section reviews the existing research on the relationships between the remaining variables (days, Tmax, pharyngeal discomfort, cough, dyspnea, altered mental status) and CAP itself. Integrating findings from previous studies will enhance the interpretability of our model and lay the groundwork for future research on risk factors.

***Days as a predictor***

The metric "days since onset" captures the heterogeneity among different patients in terms of disease progression. This is particularly critical in elderly patients. Due to immunosenescence and a higher prevalence of comorbidities, elderly individuals often experience more rapid clinical deterioration. Furthermore, the atypical and subtle clinical presentation of disease progression in the elderly is a significant reason for the prolonged days since onset. The latest Chinese guidelines on geriatric pneumonia indicate that elderly pneumonia patients often have an insidious onset and lack typical respiratory symptoms[8]. This leads to delayed medical consultation (prolonged days since onset), which in turn contributes to severe disease progression (development into severe pulmonary infection). Therefore, the value of days since onset in the diagnosis of pneumonia, especially among the elderly population, should be emphasized. This is precisely the reason why our study constructed an interaction term between age and this variable.

***Tmax*** ***as a predictor***

Maximum body temperature provides a quantitative assessment of the pathophysiological phenomenon of fever and serves as a core marker of the body's systemic inflammatory response to infection. A previous large cohort study demonstrated that a body temperature >37.8°C is the most important and independent risk factor for radiographically confirmed pneumonia[9]. However, body temperature itself, as a continuous variable reflecting the degree of fever, holds significant clinical value beyond just diagnostic purposes, including substantial prognostic value, as evidenced by its inclusion in scoring systems like the PSI mentioned in the main text. A study focusing on sepsis similarly illustrated how trajectories of temperature variables can delineate distinct prognostic sub phenotypes[10]. This suggests that for the comprehensive course of CAP management—from diagnosis through subsequent treatment and prognostic assessment—there is a future need to establish dynamic prediction models based on variables such as body temperature.

***Pharyngeal discomfort as a predictor***

Pharyngeal discomfort is a clinical manifestation of upper respiratory tract infection. In our study, patients presenting with pharyngeal discomfort exhibited a lower probability of having CAP. In the main text's discussion, we integrated certain theories from TCM, suggesting that this symptom may serve as a differentiating factor between upper and lower respiratory tract infections. Patients experiencing pharyngeal discomfort might still be in the early, localized, and controllable stage of infection, which has not yet progressed to deeper pulmonary involvement, i.e., pneumonia. As mentioned previously in the supplementary materials, existing CAP diagnostic criteria insufficiently address clinical symptoms, potentially due to the extensive diversity of early disease manifestations. However, in the process of exogenous infections, particularly viral infections, leading to CAP, their interaction with the pre-existing microbial environment encompassing the oral cavity, pharynx, trachea, and lungs is inevitable. For instance, Streptococcus pneumoniae is a primary bacterial pathogen for pneumonia in China, and viral causes account for a high proportion of CAP cases. This implies that S. pneumoniae, colonizing the upper respiratory tract such as the oral cavity and pharynx, is highly likely to cause more severe pulmonary infection through interaction with exogenous viral infection[11]. A cohort study tracked changes in the oral and pharyngeal microbial ecology throughout the entire disease course in CAP patients[12]. In the future, initiating detailed research from both clinical and mechanistic perspectives on the upper respiratory microbiome and early symptoms of respiratory infections might mark the beginning of new scientific discoveries regarding diagnosis and treatment.

***Cough as a predictor***

Coughing itself is a physiological protective reflex that helps expel sputum and foreign materials from the airways, forming part of the airway clearance mechanism. Acute cough often indicates the presence of a respiratory tract infection, which is why it is included among the symptoms considered in the diagnostic criteria for CAP. While the presence of cough alone can aid in diagnosis, it does not reflect the severity of the condition. For example, cough is not PSI or the CURB-65 score, which are used to assess the severity of pneumonia. Conversely, in the diagnostic process for acute cough, the Chinese primary care cough diagnosis and management guidelines emphasize that excluding pneumonia through imaging is a critical step[13]. Therefore, whether to address the cough itself or to diagnose or rule out pneumonia, patients with acute cough require further screening and examinations to establish a clear diagnosis. This is precisely what is lacking in primary care institutions and among patient populations in China, leading to the widespread misuse of antibiotics[14]. Furthermore, the symptoms of cough and sputum production hold significant potential research value for pathogen diagnosis. For instance, Dunne OM investigated the pathways and mechanisms by which rhinovirus infection induces highly sensitive cough[15]. This suggests that the manifestations of cough and sputum may reflect the pathogenic mechanisms of different pathogens, and in-depth research in this area has the potential to enable pathogen-specific diagnosis based on symptoms and biomarkers.

***Dyspnea as a predictor***

Dyspnea is a key symptom reflecting impaired respiratory function and disease severity in patients with Community-Acquired Pneumonia (CAP), indicating significant compromise in the physiological processes of ventilation and oxygenation. Subjectively, patients often describe dyspnea as chest tightness or breathlessness; objectively, it manifests as decreased peripheral oxygen saturation, increased respiratory rate, or in some cases, abnormally slow breathing. As early as the CURB-65 scoring system, a respiratory rate over 30 breaths per minute—a quantifiable indicator of dyspnea—was established as a critical criterion for identifying severe pneumonia. However, accurately measuring respiratory rate is not only challenging for patients to perform themselves, but elderly pneumonia patients may present with "silent hypoxia," a phenomenon noted in a retrospective study of critical COVID-19 cases[16]. Consequently, while the presence of dyspnea in an individual patient might readily suggest a diagnosis of pneumonia, especially severe CAP, relying solely on overt symptoms and signs of dyspnea is insufficient for effective screening across a broader patient population. This conclusion is further supported by the SHAP analysis of our model, which identified dyspnea as making the smallest diagnostic contribution. The staged, multi-parameter predictive model proposed in our study, which integrates various indicators and considers interaction effects, embodies precisely this approach—one that does not over-rely on typical severe symptoms.

***Altered mental status as a predictor***

Altered mental status, often described as a delirious state, is an extremely abnormal clinical manifestation, regardless of its association with pneumonia. In the context of pneumonia diagnosis, it is a classic presentation of SCAP. Conversely, pneumonia itself is a significant risk factor for the development of delirium[17]. In our study, altered mental status showed a clear positive correlation with advanced age (**Figure S4**), making further discussion within the context of elderly patients more clinically valuable. A risk factor study indicated that 17.3% of the elderly population presenting to the emergency department experienced delirium, which was associated with high mortality[18]. Research on hospitalized elderly COVID-19 patients demonstrated that delirium can lead to long-term cognitive impairment and decline, even after recovery from the acute infection and hospitalization[19]. Chinese delirium guidelines also identify advanced age as the primary predisposing factor for delirium and emphasize that the most crucial aspect of delirium management is etiological treatment[20]. Therefore, for patients with delirium, the timely identification and diagnosis of an underlying cause such as pneumonia, followed by prompt and active treatment, holds significant value for preserving cognitive function and controlling overall mortality. This underscores the importance of our pneumonia screening model for the elderly patient population.

**9. Reference**

1. Lopez-Ayala P, Riley RD, Collins GS, Zimmermann T. Dealing with continuous variables and modelling non-linear associations in healthcare data: practical guide. BMJ. 2025;390:e082440.
2. Fan Y, Liu W, Wan R, et al. Efficacy and safety of yinqiao powder combined with western medicine in the treatment of pneumonia: A systematic review and meta-analysis. Complement Ther Clin Pract. 2021;42:101297.
3. Liu J, Yang W, Liu Y, et al. Combination of Hua Shi Bai Du granule (Q-14) and standard care in the treatment of patients with coronavirus disease 2019 (COVID-19): A single-center, open-label, randomized controlled trial. Phytomedicine. 2021;91:153671.
4. Zhao L, Tian C, Yang Y, et al. Practice and principle of traditional Chinese medicine for the prevention and treatment of COVID-19. Front Med. 2023;17(6):1014-1029.
5. Wang C, Cao B, Liu QQ, et al. Oseltamivir compared with the Chinese traditional therapy maxingshigan-yinqiaosan in the treatment of H1N1 influenza: a randomized trial. Ann Intern Med. 2011;155(4):217-225.
6. Bai Y, Liu T, Zhang S, et al. Traditional Chinese Medicine for Viral Pneumonia Therapy: Pharmacological Basis and Mechanistic Insights. Int J Biol Sci. 2025;21(3):989-1013.
7. World Health Organization. WHO international standard terminologies on traditional Chinese medicine. World Health Organization. 2022. Accessed 26, October 2022. https://apps.who.int/iris/handle/10665/352306.
8. Chinese Geriatrics Society Respiratory Branch. Expert consensus on clinical diagnosis and treatment of geriatric pneumonia (2024 edition) [J]. Chinese Journal of Tuberculosis and Respiratory Diseases, 2025,48(01):18-34.
9. Moore M, Stuart B, Little P, et al. Predictors of pneumonia in lower respiratory tract infections: 3C prospective cough complication cohort study. Eur Respir J. 2017;50(5):1700434.
10. He S, Lee J, Mitragotri S, et al. Exploring temperature trajectories in emergency department sepsis patients American Journal of Emergency Medicine. 2025; 95:235-242.
11. Li ZJ, Zhang HY, Ren LL, et al. Etiological and epidemiological features of acute respiratory infections in China. Nat Commun. 2021;12(1):5026. Published 2021 Aug 18.][ Zubiria-Barrera C, Yamba LY, Klassert TE, et al. Profiling the nasopharyngeal Microbiome in patients with community-acquired pneumonia caused by Streptococcus pneumoniae: diagnostic challenges and ecological insights. Med Microbiol Immunol. 2025;214(1):19.
12. Hong L, Suo L, Chang K, et al. Longitudinal profiling of host response and oropharyngeal respiratory microbiome reveals dynamic alterations during recovery from community-acquired pneumonia. Biosaf Health. 2025;7(3):152-165.
13. Chinese Medical Association, et al. Chinese guidelines for the diagnosis and management of cough in primary care (2024). Chinese Journal of General Practitioners, 2024, 23(8):793-812.
14. Fan Z, Zhang Y, Wu Y, Yin J, Sun Q. Public understanding and misuse of antibiotics in China: insights from a comprehensive national survey. Lancet Reg Health West Pac. 2025; 62:101682.
15. Dunne OM, Roe NAM, Mousnier A, et al. Neuroinflammatory Consequences of Rhinovirus Infection in Human Epithelial and Neuronal Models. Lung. 2025;203(1):93.
16. Alamé K, Lemaitre EL, Abensur Vuillaume L, et al. Silent Hypoxemia in the Emergency Department: A Retrospective Cohort of Two Clinical Phenotypes in Critical COVID-19. J Clin Med. 2022 Aug 27;11(17):5034.
17. Zhang Yueyang, Chen Yahong, Song Yuqing, et al. Research progress on delirium in patients with severe pneumonia. Chinese Journal of Respiratory and Critical Care Medicine, 2021, 20(8):591-597.
18. Han JH, Shinar A, Eden S, et al. Delirium in the emergency department: an independent predictor of death within six months. Annals of Emergency Medicine. 2010;56(3):244-252.e1.
19. Minnema J, Tap L, Abbink EJ, et al. Trajectories After In-Hospital Delirium: Long-Term Changes in Frailty and Cognition After COVID-19. Journal of the American Medical Directors Association. 2025;26(8):105591.
20. Chinese Society of Neurology, Neuropsychology and Behavioral Neurology Group, Chen Haibo, Wang Kai, Li Shuhua. Chinese expert consensus on diagnosis and treatment of delirium in general hospitals (2021). Chinese Journal of Geriatrics, 2021, 40(10):1226-1233.
